# Characterization and thermoelectric performance of mechanochemically synthesized Cu–Ag–Se sample series

**DOI:** 10.1039/d6na00307a

**Published:** 2026-08-07

**Authors:** Dáša Drenčaková, Marcela Achimovičová, Jiří Navrátil, Matej Baláž, Erika Tóthová, Tomáš Plecháček, Mária Bali Hudáková, Stanislav Šlang, Viktor Puchý, Petr Levinský, Ludvík Beneš, Pavlína Ruleová

**Affiliations:** a Institute of Geotechnics, Slovak Academy of Sciences Watsonova 45 Košice Slovakia achimovic@saske.sk; b Faculty of Materials, Metallurgy and Recycling, Technical University of Košice Letná 1/9 Košice Slovakia; c Faculty of Chemical Technology, University of Pardubice Studentská 573 Pardubice Czech Republic; d Institute of Materials Research, Slovak Academy of Sciences Watsonova 47 Košice Slovakia; e Institute of Physics, Czech Academy of Sciences Cukrovarnická 10 Praha Czech Republic

## Abstract

The Cu_2−*x*_Ag_*x*_Se potential thermoelectrics series (*x* = 0.4–1.8) is prepared in a planetary ball mill by high-energy milling of Cu, Ag, and Se powders. After only 7 min of mechanochemical synthesis, the products are analyzed by X-ray diffraction, particle size distribution, specific surface area, X-ray fluorescence analysis, scanning electron microscopy, and thermal analysis. Unlike conventional high-temperature or multi-step syntheses, this rapid mechanochemical method yields phase-pure CuAgSe along with controlled excess metal selenides, thereby enabling a direct correlation among composition, reversible nanoscale ionic rearrangements, and charge-transport behaviour. XRD shows the same multiphase composition in powders and densified pellets, indicating no significant changes during sintering. The BET and PSD values remain similar across all compositions, while SEM reveals two distinct phases whose identities are verified by EDX. DTA shows composition-dependent phase transitions that govern the complex thermal behavior of Cu_2−*x*_Ag_*x*_Se. Tuning the Cu : Ag ratio induces p-to n-type inversion and mixed carrier transport. By systematically mapping how Cu–Ag substitution tunes dual phase transitions, carrier-type inversion (p → n), and mixed electron–hole transport, this work provides fundamentally new mechanistic insight into the interplay between superionic mobility and thermoelectric performance. Remarkably, high *ZT* values up to 1.47 are achieved without annealing or microstructural engineering, demonstrating that rapid, green mechanochemical synthesis can produce high-performance Cu–Ag–Se thermoelectrics comparable to state-of-the-art methods. The highest *ZT* of 1.47@574 K is reached for p-type Cu_1.6_Ag_0.4_Se, and for n-type Cu_0.6_Ag_1.4_Se, 0.73@570 K after the phase transition.

## Abstract 2

Cu–Ag–Se samples showed stable two-phase structures and a shift from p-type to n-type conductivity with increasing Ag content. Their *ZT* values reached up to 1.47 and 0.73, matching the performance of materials prepared by conventional methods.

## Graphical abstract

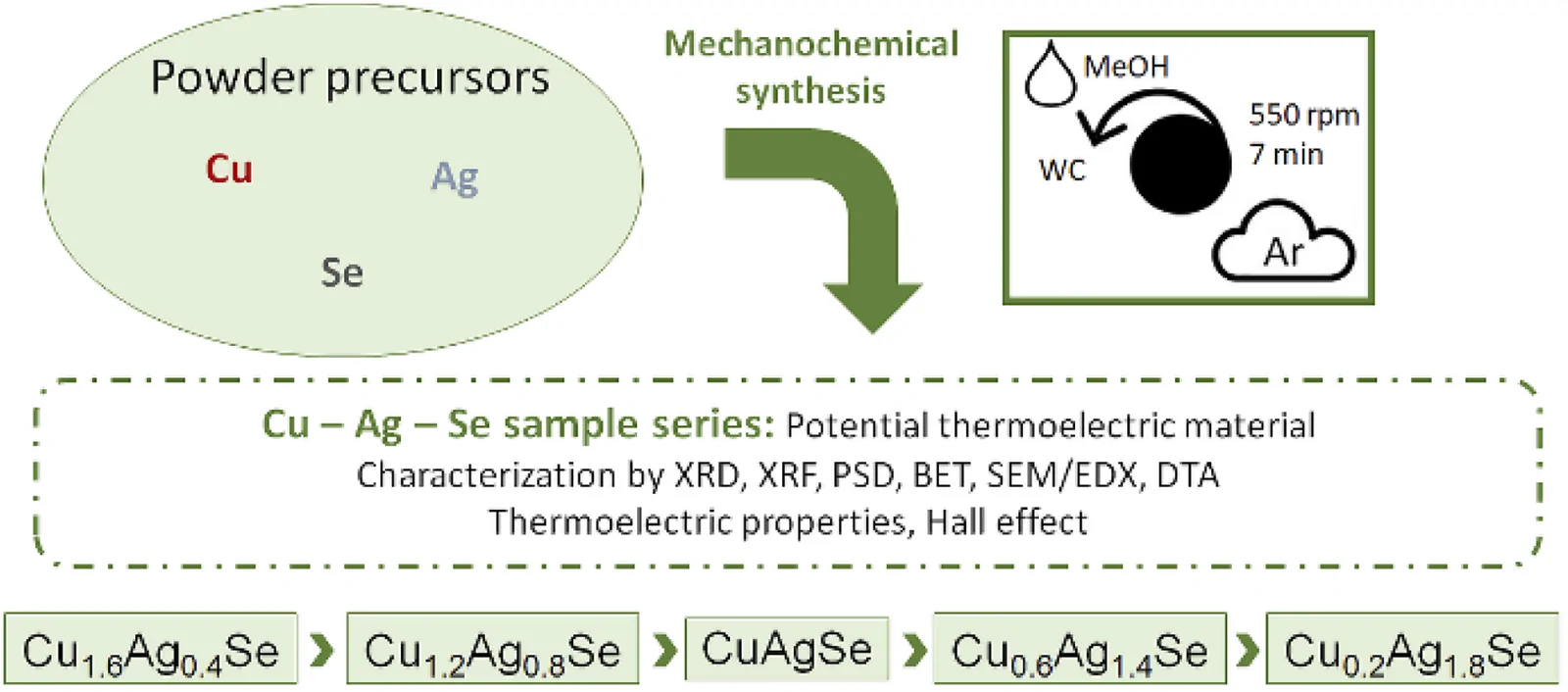

## Introduction

Transition metal chalcogenides generally offer a wide range of applications. Concretely, copper(i) silver selenide, CuAgSe, has potential for use in photocatalysis,^1^ in biomedicine due to its antibacterial activity,^2^ and in thermoelectrics, either as a traditional solid-state thermoelectric material^3–6^ or flexible thermoelectric devices (F-TED).^7–11^ The latter has the potential to be used in wearable devices as a self-powered and maintenance-free power supply for flexible electronics. The appeal of CuAgSe compounds is attributable not only to their excellent thermoelectric properties but also to their unusual mechanical properties – specifically, flexibility and ductility – which are essential for their use in F-TEDs. These properties can be effectively tailored by introducing suitable secondary phases in the matrix component.^12–14^ The employment of ball milling technology in the fabrication of F-TEDs appears to be a promising avenue for research, as it enables a one-pot ball-milling method for the simultaneous and efficient preparation/synthesis of active thermoelectric materials/composites, which can subsequently be utilised in ink formulations for direct application in screen printing or 3D printing processes in the F-TED production line.^15^

In principle, thermoelectric materials are capable of direct conversion of thermal energy into electrical energy and reversibly convert electrical energy into heat. The thermoelectric figure of merit *ZT*, which allows comparison of various thermoelectric materials, can be calculated according to eqn (1):

1 *ZT* = (*σS*^2^/*κ*)*T*

where *σ* is electrical conductivity, *S* is Seebeck coefficient, *κ* is thermal conductivity, and *T* is temperature in K.^16^ An overview of reported CuAgSe *ZT*_max_ values is provided in the Discussion section below.

CuAgSe, a synthetic analogue of natural mineral eucairite, undergoes a reversible phase transition in the temperature range 463–468 K,^17^ connected with a crystal structure change from tetragonal^18^ or orthorhombic^17^ low-temperature β-CuAgSe to cubic high-temperature α-CuAgSe with superionic behaviour.^19^ An interesting fact is that Cu_2_Se is a p-type semiconductor,^20^ Ag_2_Se is n-type,^21^ while CuAgSe exhibits switching between these two types during phase transition.^22^

In the literature, the most common preparation method of CuAgSe is solid-state synthesis.^23–29^ The main advantage of this method is the high purity of the desired product, but it requires temperatures up to 1373 K kept for several hours, making the whole process time- and energy-demanding.^30^ The chemical precipitation method can also be utilized to prepare CuAgSe,^1,2,31,32^ but it is rarely used for thermoelectric applications.^22,33^ The process occurs at room temperature, although it involves more steps and requires additional solvent waste disposal. Other methods of CuAgSe synthesis include ball milling,^3,6,34,35^ the conversion of Cu_2−*x*_Se and silver trifluoroacetate to CuAgSe nanoparticles, with a total procedure time exceeding 24 h,^1^ and manual mixing of elemental powders in a mortar with a pestle for 10 min, followed by spark plasma sintering.^36^ Jia *et al.* prepared CuAg_1+*x*_Se by electric field-assisted mass transfer from Cu, CuSe, and Ag_2_Se;^4^ by solvothermal synthesis, porous (CuAg)_2_Se was prepared;^37^ and by magnetron sputtering, Cu–Ag–Se films with different Ag content were deposited on a Si substrate.^7^

Mechanochemical synthesis of CuAgSe is a beneficial green chemistry method.^38^ It is a one-step, one-pot, solvent-free reaction that occurs under ambient pressure and room temperature. Various conditions can influence the course of mechanochemical synthesis, such as the exact mill type, the materials of the milling chamber and balls, the duration of the whole milling process, the temperature reached in the milling chamber, milling speed, ball-to-powder weight ratio, and type of atmosphere in the milling chamber.^39^ For the first time in 1997, Ohtani^34^ prepared orthorhombic CuAgSe by milling in a planetary ball mill for 1 h; later, in 2018, Olvera *et al.*^6^ used up to 16 h of milling time to prepare mixed orthorhombic and tetragonal CuAgSe. Recently, in 2023, Yu *et al.*^3^ also reported a 16 h milling time; however, in our previous study,^40^ only a 7 min milling time was needed due to the usage of a tungsten carbide milling chamber, unlike previous authors who used an agate milling chamber. The simple, rapid mechanochemical synthesis of a series of nanostructured Cu_2−*x*_Ag_*x*_Se materials, along with their thermoelectric performance, has not yet been reported.

In the present study, a series of nanostructured Cu_2−*x*_Ag_*x*_Se samples with different Cu : Ag ratios were mechanochemically synthesized in a planetary ball mill in just 7 min, under conditions previously reported for the eucairite synthesis.^40^ The physicochemical properties of the products, such as phase and chemical compositions, morphology, thermal stability and reversibility, and thermoelectric properties, were studied using various techniques. This work delivers new mechanistic insight into how superionic mobility governs thermoelectric performance by systematically tracing how Cu– Ag substitution modulates dual phase transitions, drives p-to-n carrier inversion, and enables mixed electron–hole transport.

## Experimental

The mechanochemical synthesis of the Cu_2−*x*_Ag_*x*_Se sample series was performed in a planetary ball mill (Pulverisette 6, Fritsch, Germany). For the synthesis, elemental powders of copper (99%, 116 µm, Nippon Atomized Metal Powders Corp., Japan), silver (99,9%, 125 µm, Thermo Scientific Chemicals), and selenium (99,5%, 74 µm, Acros Organics) were used. The amounts of each precursor were calculated to yield 5 g of Cu_2−*x*_Ag_*x*_Se product with various chemical compositions corresponding to *x* = 0.4, 0.8, 1.0, 1.4, and 1.8. The following milling conditions were applied: tungsten carbide (WC) milling chamber with volume 250 mL loaded with 50 pieces of WC-balls (10 mm in diameter), ball-to-powder weight ratio 73 : 1, inert Ar atmosphere, 1 mL of methanol as additive, rotation speed 550 rpm, and milling time *t*_M_ = 7 min. The spark plasma sintering (SPS) method was used to densify the powdered samples in the furnace HP D10-SD (FCT Systeme GmbH, Germany). The Cu_2−*x*_Ag_*x*_Se powder was placed into a graphite die (inner diameter 10 mm) and sintered into a round pellet about 2 mm thick at 673 K under 64 MPa (heating rate 100 K min^−1^), with a holding time of 10 min in an Ar atmosphere. Qualitative X-ray diffraction (XRD) analysis of powders was performed using a diffractometer D8 Advance (Bruker, Germany) in Bragg–Brentano geometry with Cu_Kα_ radiation (*λ* = 0, 154 nm) at 40 mA and 40 kV. Phase identification was carried out using the DiffracEVA software (Bruker, Germany) and the JCPDS PDF database. Rietveld refinements of the XRD data of the as-prepared samples were performed using the TOPAS Academic program.^41,42^ For quantitative elemental analysis, the µ-XRF spectrometer M4 Tornado (Bruker, Germany) was used, equipped with polycapillary X-ray optics. Particle size distribution (PSD) was measured using the Camsizer X2 particle size and shape analyzer (Retsch, Germany) with dual camera technology and an X-dry sample feeder. The measuring range was set to 0.01–5 mm for Cu_0.2_Ag_1.8_Se and 0.01–1 mm for other Cu_2−*x*_Ag_*x*_Se samples. Using the low-temperature nitrogen adsorption method in the Gemini 2360 sorption apparatus (Micromeritics, USA), the Brunauer–Emmett–Teller (BET) specific surface areas, *S*_BET_, were determined.

Scanning electron microscopy (SEM) analysis was performed using a LYRA 3 SEM microscope (TESCAN, Czech Republic), equipped with a secondary electrons detector (SE), a backscattered electrons detector (BSE), and an energy-dispersive X-ray spectroscopy (EDX) detector (Oxford Instruments, UK). The observed surfaces of the samples were the unpolished sides of the pellets after cutting them into smaller pieces.

The thermal stability of the samples was investigated by TG/DTA measurements using a thermal analyzer STA 449 F3 Jupiter (Netzsch, Germany). The measurements were carried out over the temperature range of 298–523 K at a heating rate of 10 K min^−1^ under an argon atmosphere (purity 99.999%) with a constant flow rate of 40 mL min^−1^. The thermal program consisted of a heating cycle (298–523 K), followed by a 10 min isothermal hold at 523 K, and a subsequent cooling cycle (523–298 K). Al_2_O_3_ crucibles were used both for the sample measurements and as the reference material.

The four-terminal method was used to measure electrical conductivity, *σ*, of a round pellet on the LSR-3 instrument (Linseis, Germany) from 300 to 583 K. The Seebeck coefficient, *S,* was measured *via* the static DC method using the LSR-3, and electrical conductivity was measured simultaneously. The measurements were performed in a helium atmosphere under 0.1 bar overpressure. The Seebeck coefficient, *S*, together with electrical conductivity, *σ*, were also measured under liquid N_2_ from 90 to 300 K using a home-made apparatus. The thermal diffusivity, *k*, was measured from 173 to 573 K on a round pellet using an LFA 467 instrument (Netzsch, Germany). The thermal conductivity *κ*, was calculated according to the relation *κ* = *k* × *c*_p_ × *ρ*, where *k* is the thermal diffusivity, *c*_p_ is the heat capacity, and *ρ* is the sample density. Super-alloy Inconel was used as the standard for heat capacity. Finally, the thermoelectric figure of merit, *ZT,* was calculated according to eqn (1). To determine Hall coefficients, *R*_H_, sample pellets were cut into geometrically suitable pieces using a diamond wire saw. The Hall coefficient *R*_H_ was measured over a temperature range from room temperature to 586 K or higher in an Ar atmosphere in a home-made cell. An alternating current with a frequency of 1020 Hz and a stationary magnetic field with an induction of 0.4 T were applied in the method. Welded platinum-wire voltage contacts and pressure mechanical current contacts were used.

The measurement uncertainties are ∼4% for *σ*, ∼3% for *S* and ∼6% for *κ*. The accumulated error for *ZT* is thus ∼16%. The uncertainty in *R*_H_ is believed to be less than 10%.

## Results and discussion

### Physicochemical properties of Cu_2−*x*_Ag_*x*_Se sample series

**Fig. 1:**
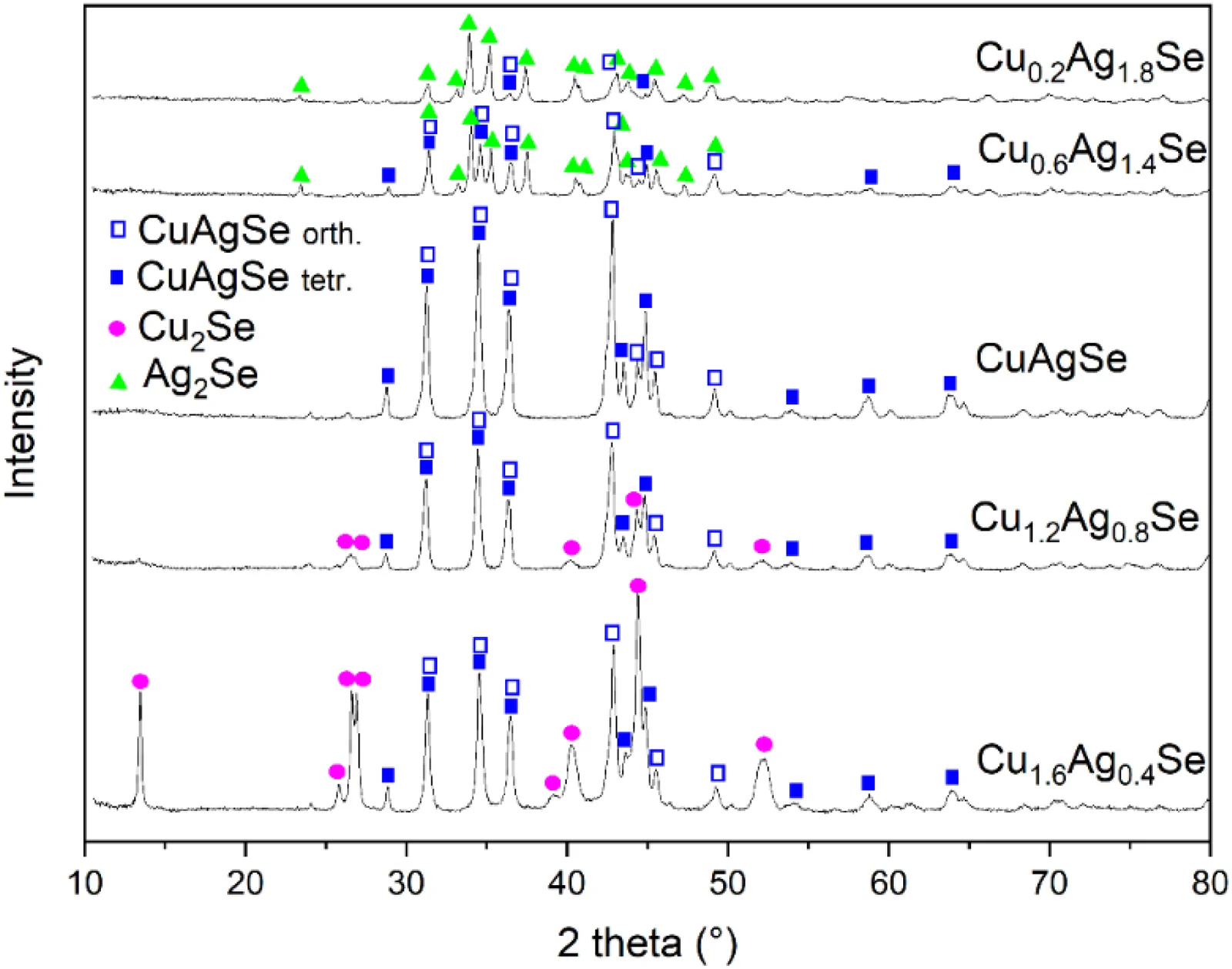
XRD patterns of milled Cu/Ag/Se mixtures with different compositions after mechanochemical synthesis.

**Fig. 2:**
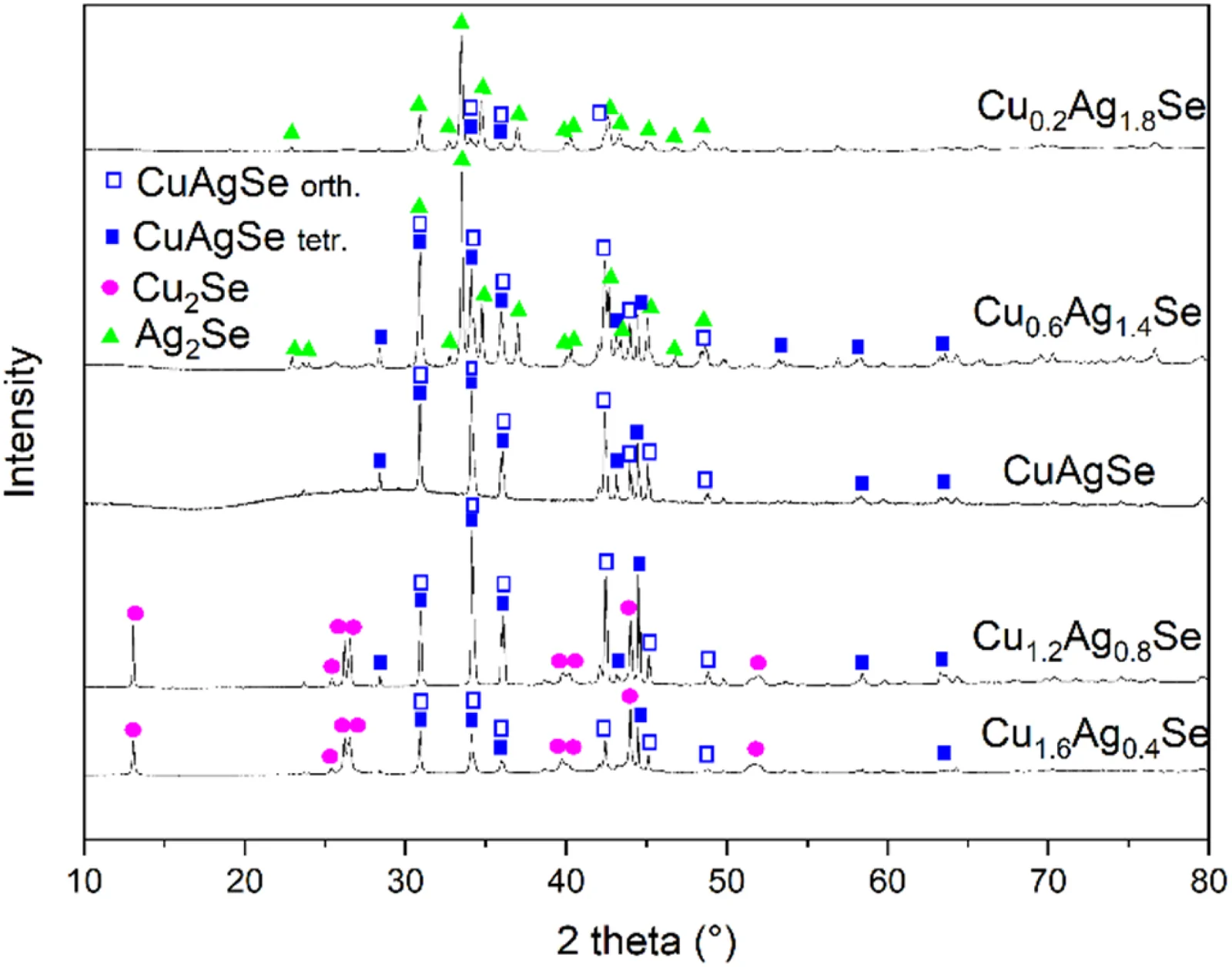
XRD patterns of mechanochemically synthesized Cu_2−*x*_Ag_*x*_Se after SPS.

XRD analysis was performed on samples directly after 7 min of mechanochemical synthesis (identified as optimal time in our previous study^40^), in powdered form (Fig. 1), and after SPS consolidation in pellet form (Fig. 2). The main difference between the XRD patterns of both forms is the peak shapes: the powders' peaks are broader, whereas the pellets' peaks are sharper. However, the identified phases remain unchanged, ruling out any significant changes in the Cu_2−*x*_Ag_*x*_Se crystallographic structure and phase transformation after SPS. Although the phase composition remains unchanged, SPS markedly modifies the microstructure. Rapid Joule heating, pulsed current, and applied pressure reduce porosity, improve grain–grain contact, and induce partial grain growth. These changes lower grain-boundary density, enhancing carrier mobility, while the modified grain size and densification influence phonon scattering and thus the lattice thermal conductivity.^43–45^

In both Fig. 1 and 2, CuAgSe is indicated as a mixture of two very similar phases: an orthorhombic (00-010-0451) and a tetragonal (01-089-3935). Samples with *x* = 0.4 and 0.8 represent mixtures of ternary CuAgSe and binary Cu_2_Se (00-047-1448), respectively, reflecting changes in composition as evidenced by more intense Cu_2_Se peaks in Cu_1.6_Ag_0.4_Se. By contrast, Cu_2−*x*_Ag_*x*_Se with *x* = 1.4, 1.8, contain CuAgSe together with orthorhombic Ag_2_Se (00-024-1041). The results of Rietveld analysis, revealing quantitative phase compositions of the Cu_2−*x*_Ag_*x*_Se sample series with corresponding refinement statistics, calculated crystallite sizes (CS) in the range of 25–47 nm, and microstrains are given in Table 1. The quantitative phase analysis supports the fact that ternary CuAgSe forms in the maximum available amount, while the remaining Cu or Ag forms binary selenides according to the following equations:

2 Cu_2−*x*_Ag_*x*_Se → *x*CuAgSe + (1 − *x*) Cu_2_Se; 0 < *x* < 1

3 Cu_2−*x*_Ag_*x*_Se → (2 − *x*) CuAgSe + (*x* − 1) Ag_2_Se; 1 < *x* < 2

**Table 1:** Quantitative phase analysis results of synthesized Cu_2−*x*_Ag_*x*_Se samples with fit quality indicators, crystallite sizes, and microstrain values

Labelling of Cu_2−*x*_Ag_*x*_Se sample with various *x*	Ag_2_Se [wt%]	CuAgSe [wt%] orth./tet.	Cu_2_Se [wt%]	*R*_wp_	*R*_exp_	GOF	CS [nm]	Microstrain
Cu_1.6_Ag_0.4_Se	0	43 ± 5/0	57 ± 5	7.363	1.229	5.993	25 ± 2^a^	1.118 ± 0.07
Cu_1.2_Ag_0.8_Se	0	68 ± 4/31 ± 4	0.8 ± 0.1	5.606	1.616	3.469	40 ± 2^b^	0.689 ± 0.04
CuAgSe	0	26 ± 11/74 ± 11	0	6.622	1.550	4.273	31 ± 1^b^	0.284 ± 0.05
Cu_0.6_Ag_1.4_Se	21 ± 2	79 ± 2/0	0	5.090	1.973	2.576	35 ± 2^b^	0.482 ± 0.04
Cu_0.2_Ag_1.8_Se	82 ± 2	2 ± 0.4/16 ± 2	0	4.492	2.142	2.096	47 ± 2^c^	0.560 ± 0.02

a Calculated for Cu_2_Se phase.

b Calculated for orthorhombic CuAgSe phase.

c Calculated for Ag_2_Se phase.

Calculated values are close to theoretical ones.

**Fig. 3:**
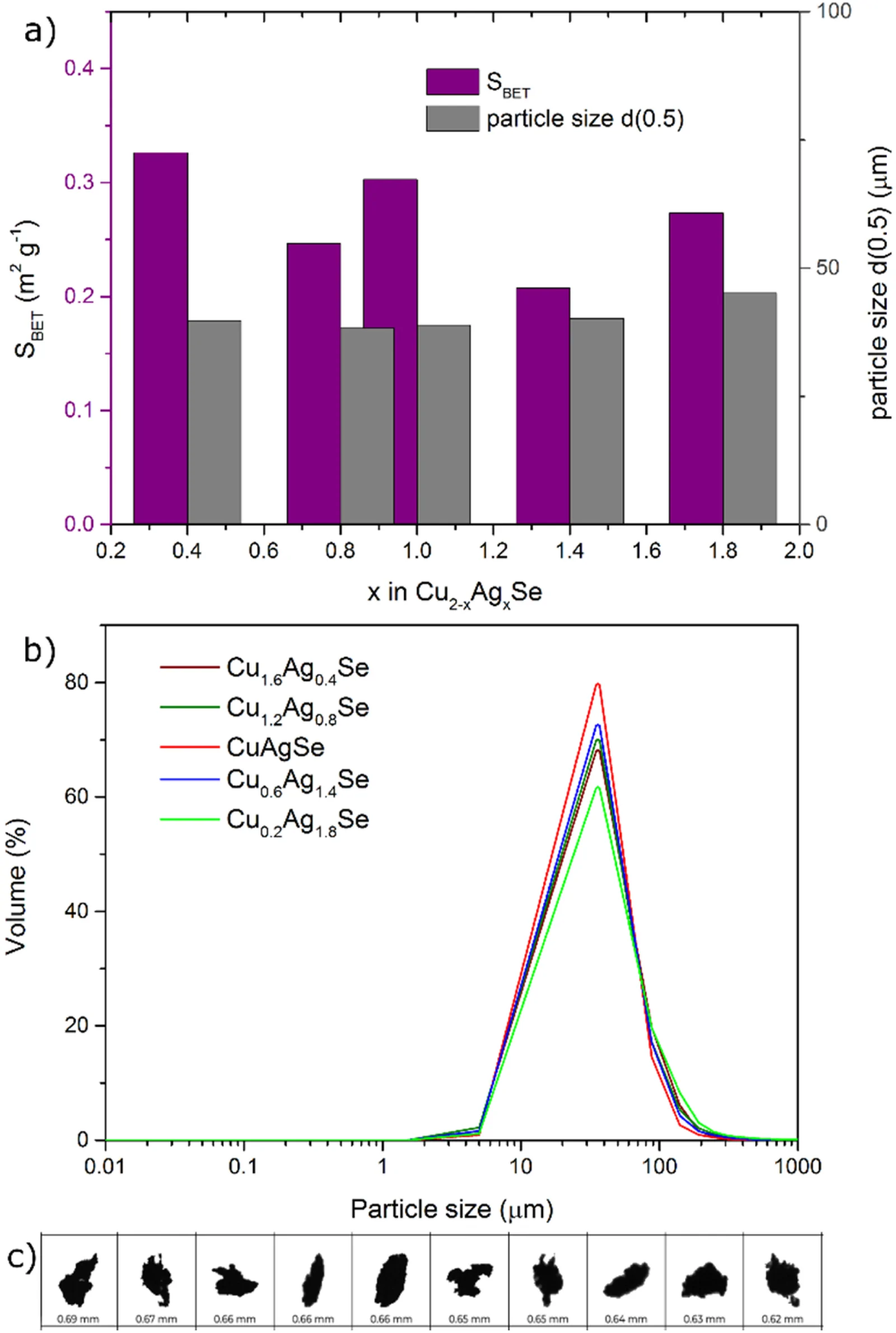
(a) Specific surface areas, *S*_BET_ and *d*(0.5) particle size of Cu_2−*x*_Ag_*x*_Se samples with various *x*, (b) PSD curves of Cu_2−*x*_Ag_*x*_Se samples, (c) selected images of various particle shapes.

Measured values of the powders' specific surface area, *S*_BET_, are comparable among samples, with no significant changes, see Fig. 3a. Therefore, after 7 min of milling under the same conditions, the Cu/Ag/Se mixtures obtained *S*_BET_ values that were close to each other, regardless of the exact chemical composition, in the range 0.21–0.33 m^2^ g^−1^. The maximum *S*_BET_ value 0.33 m^2^ g^−1^ was reached for Cu_1.6_Ag_0.4_Se. For the mean particle sizes *d*(0.5) of Cu_2−*x*_Ag_*x*_Se samples, the resulting values follow the same trend and are not influenced by chemical composition. They are similar across all samples in the range 38–45 µm. Twenty size classes were set up for PSD measurement in Fig. 3b, showing a very similar trend, with the majority of measured particles in the 10–62 µm class. Distribution curves for Cu_2−*x*_Ag_*x*_Se samples showed a monomodal particle size distribution, with the most uniform particles. Irregular shapes of agglomerated particles of the individual samples shown in Fig. 3c formed by agglomeration of original nanoparticles during mechanochemical synthesis were revealed by PSD analysis as well.

X-Ray fluorescence analysis of Cu_2−*x*_Ag_*x*_Se pellets proved changes in the Cu : Ag ratio. Comparing calculated and determined amounts to measured values in Table 2 shows a slight excess of Se in all samples, and Ag deficiency in samples with *x* = 1.4, 1.8, probably caused by the ductility of elemental Ag covering some milling balls. Elemental distribution was uniform.

**Table 2:** Chemical composition of Cu_2−*x*_Ag_*x*_Se sample series from XRF spectra

Labelling of Cu_2−*x*_Ag_*x*_Se sample with various *x*	Elements [at%]			Stoichiometric ratio		
	Cu	Ag	Se	Cu	Ag	Se
Cu_1.6_Ag_0.4_Se	53.58	12.44	33.98	1.58	0.37	1
Cu_1.2_Ag_0.8_Se	40.66	25.6	34.28	1.19	0.73	1
CuAgSe	33.00	31.82	35.17	0.94	0.90	1
Cu_0.6_Ag_1.4_Se	20.16	44.63	35.21	0.57	1.27	1
Cu_0.2_Ag_1.8_Se	7.95	56.75	35.29	0.23	1.61	1

Theoretical densities of Cu_2−*x*_Ag_*x*_Se pellets were compared to values obtained by calculation based on the dimensions and weight of each pellet. The resulting relative densities of the pellets were satisfactorily above 92% for all pellets (see Table 3). During pellet cutting, their compactness was observed. As a result, the majority of the samples were found to be well compacted within the pellet.

**Table 3:** Densities of Cu_2−*x*_Ag_*x*_Se sample series pellets. The rule of mixtures was used to estimate the density of the tablets under study

Labelling of Cu_2−*x*_Ag_*x*_Se pellet with various *x*	Theoretical density [g cm^−3^]	Observed density [g cm^−3^]	Relative density [%]
Cu_1.6_Ag_0.4_Se	7.164	6.62	92.4
Cu_1.2_Ag_0.8_Se	7.559	7.04	93.1
CuAgSe	7.750	7.42	95.7
Cu_0.6_Ag_1.4_Se	7.904	7.60	96.2
Cu_0.2_Ag_1.8_Se	8.045	7.41	92.1

**Fig. 4:**
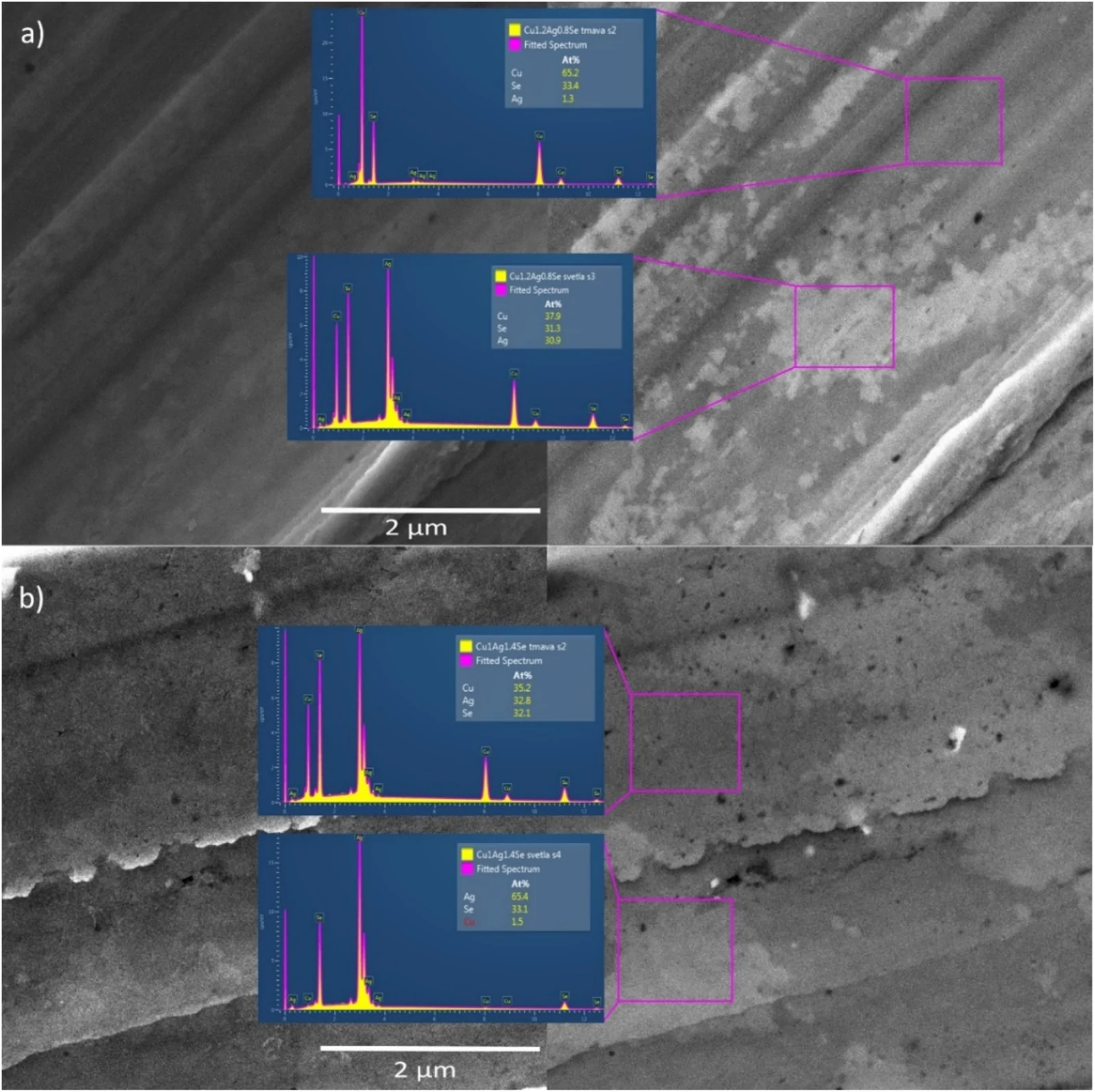
SEM microphotographs with EDX spectra of (a) Cu_1.2_Ag_0.8_Se, (b) Cu_0.6_Ag_1.4_Se; on the left side – SE images, on the right side – BSE images; the insets – EDX spectra.

Fig. 4a and b illustrate the SEM microphotographs of two selected Cu_2−*x*_Ag_*x*_Se samples – pellets with *x* = 0.8 and 1.4, with the EDX spectra of different areas of the sample. The complete results of EDX analysis for all samples in the series are presented in Table 4. SEM analysis reveals surface morphology typical for samples cut by the diamond wire saw.^46^ Different types of detectors were used. In the secondary electron (SE) image, the two coexisting phases in the samples are either invisible or barely visible. In the backscattered electron (BSE) image, however, they can be distinguished. Generally, the higher the atomic number is, the brighter the material appears in the image. Thus, for samples with *x* < 1, the bright phase corresponds to CuAgSe and the dark phase to Cu_2_Se, while for samples with *x* > 1, the bright phase was identified as Ag_2_Se and the dark phase as CuAgSe. These results are in agreement with the XRD phase analysis. Larger areas of separated phases were observed in Cu_0.2_Ag_1.8_Se, whereas the phases were more intermixed without strict boundaries in Cu_1.6_Ag_0.4_Se.

**Table 4:** Identification of various phases detected in Cu_2−*x*_Ag_*x*_Se samples by SEM/EDX

Labelling of Cu_2−*x*_Ag_*x*_Se sample with various *x*	Phase	Elements [at%]			Phase composition
		Cu	Ag	Se	
Cu_1.6_Ag_0.4_Se	Bright	39.01	30.11	30.88	CuAgSe
	Dark	63.83	2.14	34.03	Cu_2_Se
Cu_1.2_Ag_0.8_Se	Bright	38.27	30.73	31.00	CuAgSe
	Dark	63.30	4.09	32.62	Cu_2_Se
CuAgSe	One	37.60	32.00	30.40	CuAgSe
Cu_0.6_Ag_1.4_Se	Bright	1.80	64.88	33.32	Ag_2_Se
	Dark	36.06	31.78	32.17	CuAgSe
Cu_0.2_Ag_1.8_Se	Bright	1.37	67.40	31.57	Ag_2_Se
	Dark	33.99	34.95	31.06	CuAgSe

### Thermal properties of Cu_2−*x*_Ag_*x*_Se sample series

**Fig. 5:**
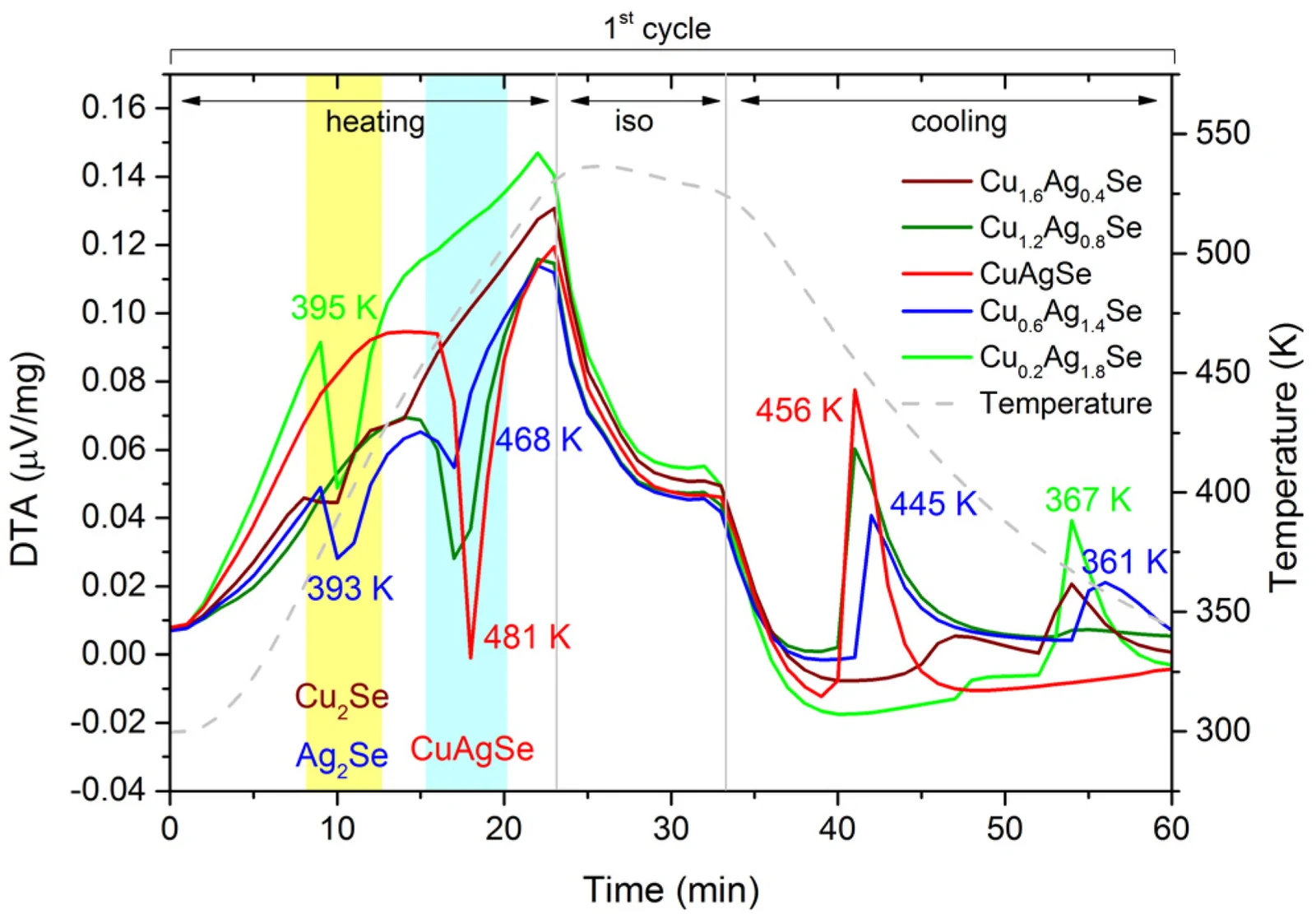
DTA curves of Cu_2−*x*_Ag_*x*_Se samples with highlighted phase transition regions of Ag_2_Se, Cu_2_Se, and CuAgSe after 1st thermal cycle.

Thermal analysis of the Cu_2−*x*_Ag_*x*_Se sample series with repeated heating/cooling cycling revealed three distinct phase transitions. Previously reported transition temperatures are 393 K for Cu_2_Se,^47^ 408 K for Ag_2_Se,^48^ and 463–468 K for CuAgSe.^17^ Thermal cycling was intentionally performed only up to 532 K, because all relevant phase transitions in CuAgSe, Cu_2_Se, and Ag_2_Se occur well below this temperature. Since the transition temperatures for the two binary selenides are close to each other, only two regions of phase transitions are color-marked in Fig. 5 for simplicity. The measured phase-transition temperatures were 395 K for Cu_2_Se, 398 K for Ag_2_Se, and 484 K for CuAgSe. Compared to the reported values in,^47^ those for Cu_2_Se were slightly higher, those for Ag_2_Se lower, and those for CuAgSe higher, though with some differences among individual samples. For instance, the CuAgSe transition temperature measured for the sample with *x* = 1.4 reached 474 K, which is closer to the reported values.^17^ As expected, the DTA curve of the Cu_2−*x*_Ag_*x*_Se sample with *x* = 1.0 lacked a peak in the first phase transition region; however, the most significant peak was observed in the second phase transition region, and was assigned to CuAgSe. On the contrary, samples with *x* = 0.4 and 1.8 exhibit the highest peaks in the first region, corresponding to Ag_2_Se or Cu_2_Se, while peaks for CuAgSe phase transition are absent in the second region. Cooling data for all samples confirmed the reversibility of the phase transitions of every component. Three-fold thermal cycling also clarified that CuAgSe, Cu_2_Se and Ag_2_Se exhibit reversible superionic transitions and do not undergo irreversible structural degradation, largely due to the intrinsic disorder-order nature of the Cu^+^/Ag^+^ sublattice (see Fig. S1 in the SI). Such mechanochemically synthesized materials show enhanced defect tolerance, mitigating microstructural fatigue during repeated heating/cooling cycles.^49^ The temperature shift of the phase-transition peaks during heating and cooling in all samples indicates increased structural disorder induced by the mechanochemical preparation.^40,50^

**Fig. 6:**
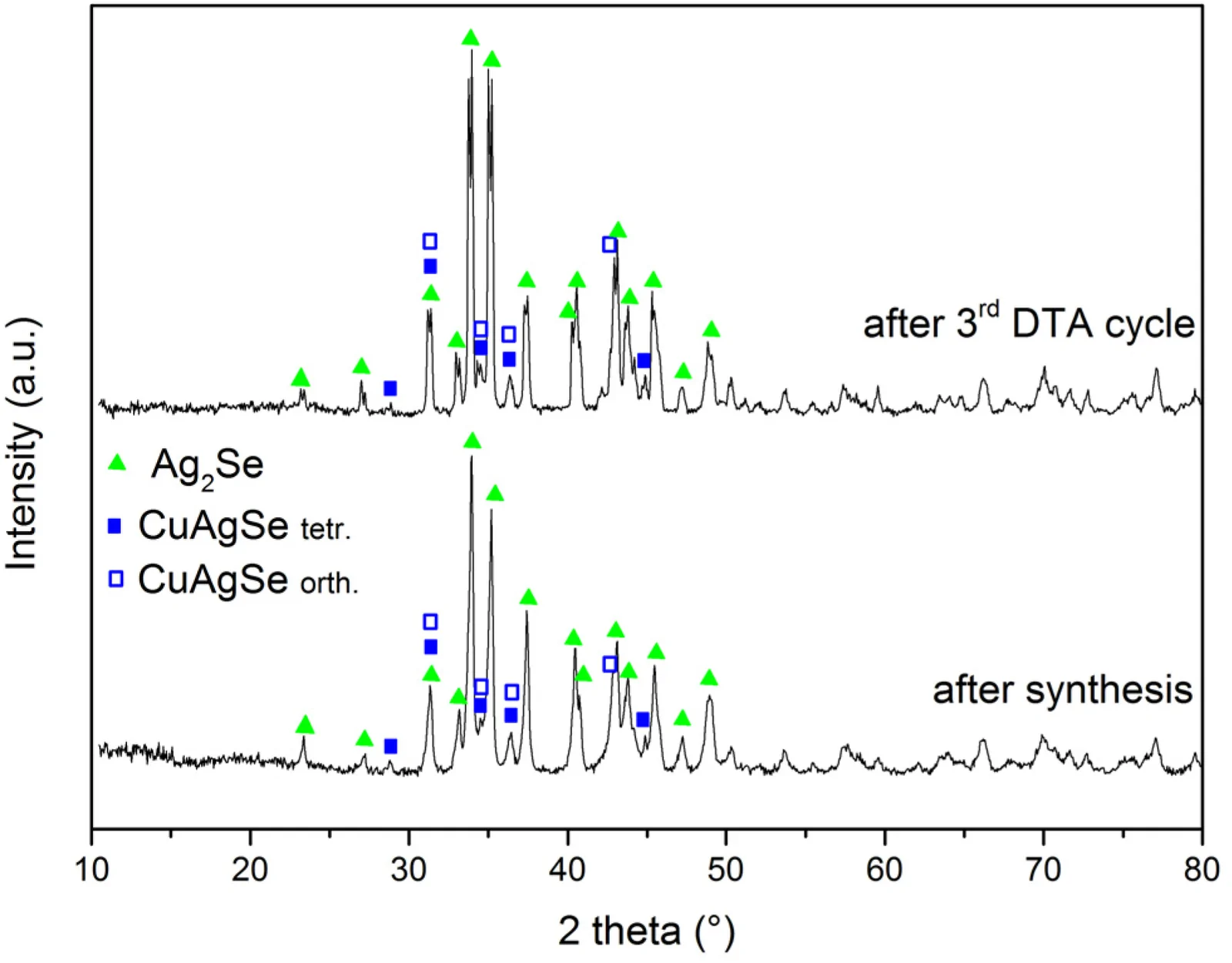
Comparison of XRD patterns of Cu_0.2_Ag_1.8_Se before and after DTA cycling.

XRD analyses of all samples in Fig. S2 (see SI) after three-fold thermal cycling showed changes only in diffraction peak intensities, not in peak positions. Thus, the identified phases remained the same and stable. To illustrate this, in Fig. 6, the peaks corresponding to each phase identified in the sample Cu_0.2_Ag_1.8_Se, with a more significantly shifted phase transition peak on the DTA curve during cooling, were marked. Recrystallization induced by thermal analysis is a likely cause of the changes in peak intensities.

### Thermoelectric properties of Cu_2−*x*_Ag_*x*_Se sample series pellets

**Fig. 7:**
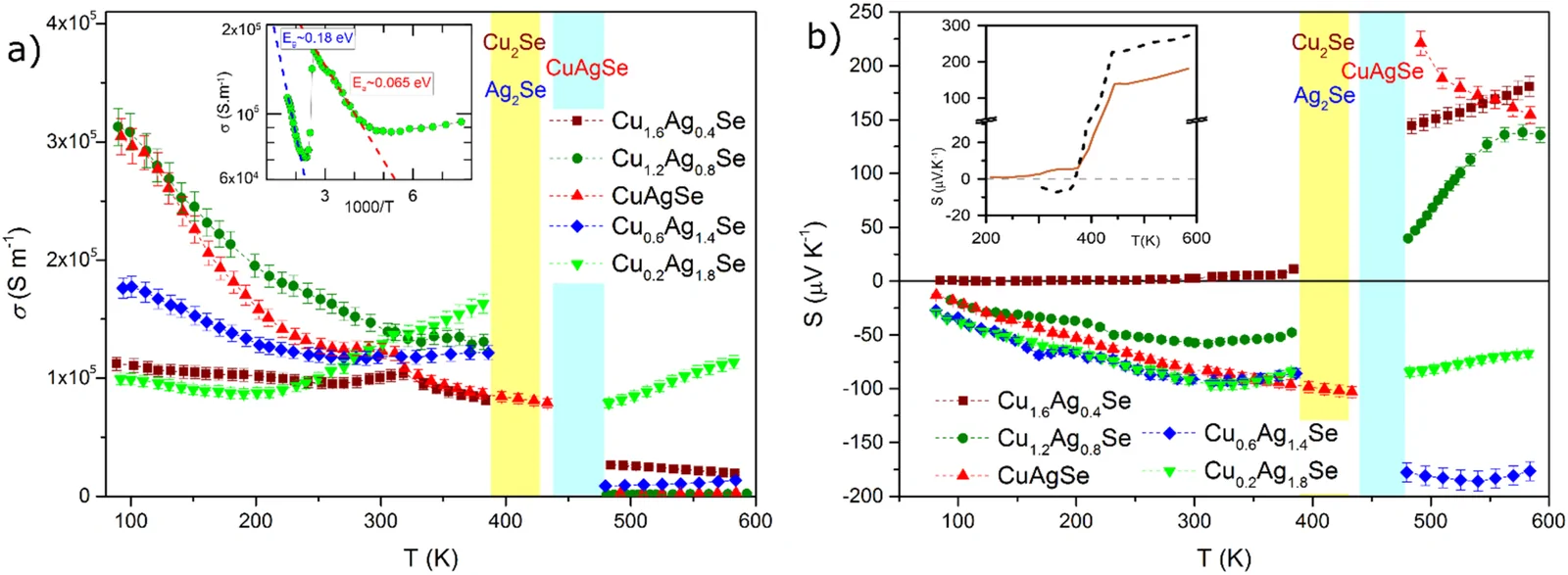
Electrical transport properties of Cu_2−*x*_Ag_*x*_Se samples, temperature-dependent (a) electrical conductivity, (b) Seebeck coefficient.

The thermoelectric properties of all Cu_2−*x*_Ag_*x*_Se pellets were studied at various temperature ranges using the methods detailed in the Experimental part. In our opinion, the studied Cu_2−*x*_Ag_*x*_Se composites should approximately consist of the maximum available amount of CuAgSe mixed with either Cu_2_Se (*x* < 1) or Ag_2_Se (*x* > 1). See eqn (2) and (3). The electrical conductivity, *σ* for all Cu_2−*x*_Ag_*x*_Se samples decreases as temperature increases (Fig. 7a), proving their metallic conduction behavior in the α-phases region. All materials exhibit rather high *σ* values ranging from approximately 1 × 10^5^ to S m^−1^ to 1.5 × 10^5^ S m^−1^ at room temperature. As the materials approach their respective phase transitions, their electrical conductivity *σ* begins to dip slightly (unlike Cu_0.2_Ag_1.8_Se). However, once they transition to their superionic high-temperature cubic structures, conductivity plummets sharply by at least one order of magnitude. This significant decline is driven, in part, by the highly dynamic nature of the superionic phases in each material.

The significantly higher *σ* values in the region where both superionic phases coexist, *i.e.* above 480 K, suggest that the Cu_2_Se phase dominates in Cu_2−*x*_Ag_*x*_Se composites with the highest Cu/Ag ratio, while the Ag_2_Se phase dominates in composites with the lowest Cu/Ag ratio. For the Cu_0.2_Ag_1.8_Se sample, we observed two areas exhibiting Arrhenius behavior in their *σ* values. Due to the dominant contribution of the Ag_2_Se component to the total *σ*, we hypothesize that this behavior is due to the activation of electrons across the band gap, *E*_g_*.* The determined *E*_g_ values for the α-phase of Ag_2_Se are 0.03 eV, and for the β-phase are 0.18 eV (see inset in Fig. 7a). These values are in accordance with literature data.^51,52^

A simple qualitative comparison of the end members of the series under study suggests that the overall conductivity of the composites (*i.e.*, Ag_2_Se/CuAgSe and Cu_2_Se/CuAgSe) can be well approximated by the rule of mixture model (ROM). The ROM is defined by the following equation: *σ*_c_ = *V*_a_*σ*_a_ + (1 − *V*_a_)*σ*_b_, where *V*_a_ is a volume fraction of the Ag_2_Se and/or Cu_2_Se in the composites, *σ* are the individual conductivities of either Ag_2_Se or Cu_2_Se (*σ*_a_) or CuAgSe (*σ*_b_). The *σ* values for the binaries were used from ref. 53 for Ag_2_Se (exactly Ag_2_Se_1.01_ for the best coincidence) and from ref. 49 for Cu_2_Se. The same values for CuAgSe were taken from this work. As Fig. S3 (see SI) shows, the ROM model provides a very good qualitative explanation of the electrical conductivity of most of the studied composites, and its results correspond to the analysis of the individual components shown in Table 1. The rather small discrepancies observed between the model and the measured values can be attributed to slight variations in the stoichiometry of the individual components constituting the composites. The higher observed difference for the Cu_1.6_Ag_0.4_Se composite containing a predominant amount of the Cu_2_Se phase is likely due to this composite consisting of grains with both p-type (Cu_2_Se) and n-type (CuAgSe) electrical conductivity. In this particular instance, p–n heterojunctions are formed at the grain boundaries, thereby giving rise to substantial contact resistance, which in turn imposes limitations on carrier mobility. This indicates that the composite's actual electrical conductivity will be considerably lower than predicted by the standard rule for mixtures.

The negative value of the Seebeck coefficient *S* within the low-temperature region (*T* < *T*_β→α_) for most of the studied materials (see Fig. 7b), indicates that the transfer of electric charge in metallic or degenerate systems is dominated by free electrons. The only exception is the Cu_1.6_Ag_0.4_Se composite, which exhibits very small positive values of the Seebeck coefficient (between +1 and +5 µV K^−1^) in this temperature range. In this case, the values reflect the fact that the composite consists of both p-type (Cu_2_Se) and n-type (CuAgSe) components, mixed in an approximately 60 : 40 molar ratio.

**Fig. 8:**
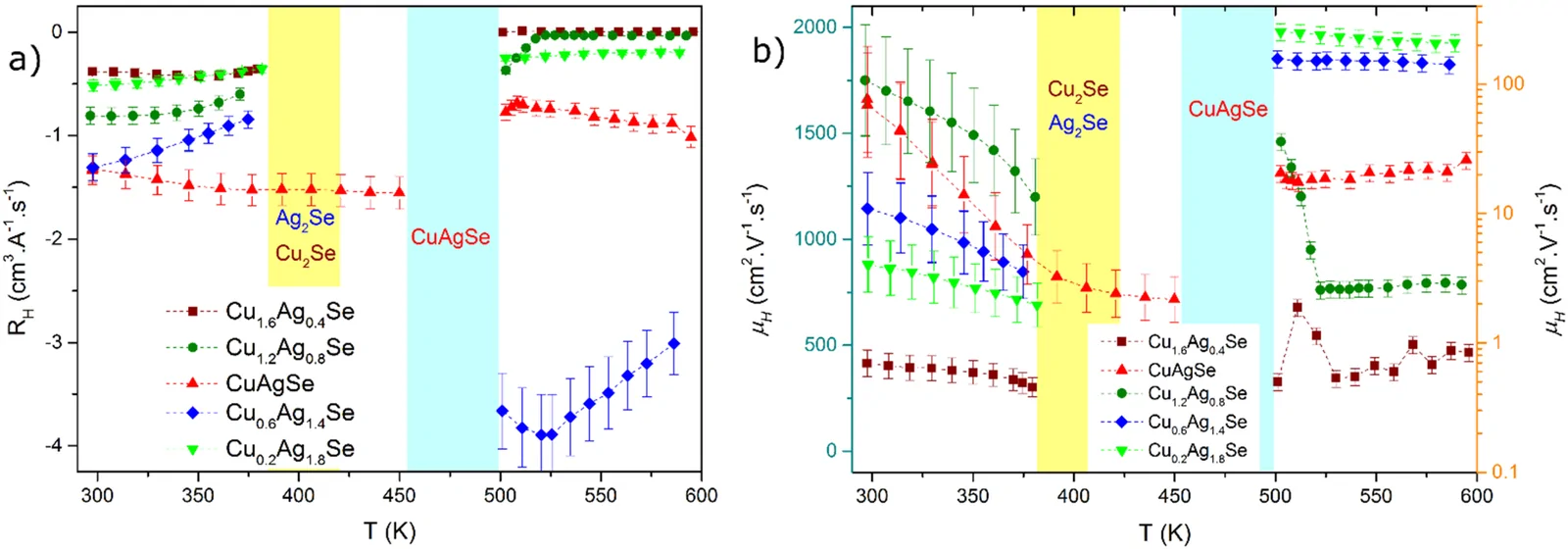
Hall effect measurement, Cu_2−*x*_Ag_*x*_Se temperature-dependent (a) Hall coefficient *R*_H_, (b) Hall mobility *µ*_H_. Left *y*-axis represents the values of *µ*_H_ up to temperatures of 470 K, while the right *y*-axis in logarithmic scale represents the values of *µ*_H_ in the β-phase.

As the temperature rises further, the components of the prepared composites undergo the previously mentioned phase transformations. The values of the Seebeck coefficient are subject to a marked change due to restructuring and alterations in the point-defect spectrum. In the case of pure CuAgSe, our previous findings^36^ demonstrated that following its phase transformation, the *S* values undergo a transition from negative to positive, whilst the Hall coefficient *R*_H_ maintains a negative sign (see Fig. 8a). According to Wang *et al.*,^30^ the observed change can be attributed to the presence of cation deficiency (Ag, Cu, or both) within the disordered high-temperature phase. Similar behavior of the *S* can be observed in the CuAgSe-rich composite with Cu_2_Se, *i.e.*, in Cu_1.2_Ag_0.8_Se. In 36, it was demonstrated that the observed difference between the opposite signs of the *S* and *R*_H_ in the case of α-CuAgSe can be explained by a two-carrier model. In this model, the observed discrepancy can be expected when the hole concentration exceeds the electron concentration, in cases where there is significantly higher mobility of electrons compared to the mobility of the present holes. For Cu_2−*x*_Ag_*x*_Se composites with a greater content of Ag_2_Se, specifically with *x*-values of 1.4 and 1.8 respectively, the total Seebeck coefficient can be expressed as an approximate weighted sum of the two components.^6^

Fig. 8a shows the temperature dependence of the Hall coefficient for the samples under investigation. To better explain the changes observed in the temperature-dependent Hall coefficient measurements of the composites under study, we summarize some properties of their individual components below. We will focus in particular on the nature of the free charge carriers involved in charge transport and the present point defects.

The low-temperature β-phase of CuAgSe has a semimetallic character and contains two types of carriers (holes and electrons). The Cu and Ag act as donors and are probably located in interstitial positions, providing numerous low-mass electrons (m* = 0.1 × *m*_0_ (ref. 54)) with extremely high mobility.^40^ Although a considerable number of holes are also present in the compound, compensating for negatively charged cation vacancies (V_Cu_ and/or V_Ag_),^30^ the extremely high electron mobility mitigates the effect of these carriers, resulting in a negative *S* value. The dominance of highly mobile light electrons is also evidenced by the fact that, when correlating experimental *S* values with the measured Hall concentration *n* = 1/(*R*_H_ × *e*) using the Mott formula4
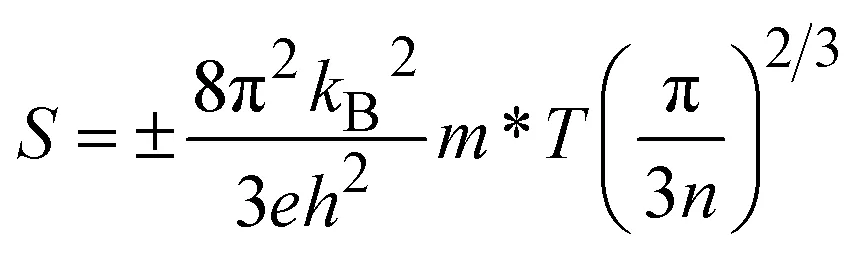


We obtained fairly good agreement in the β-phase region with the experimental data for the value m* ∼ 0.1 × *m*_0_.^54^ In the above-presented equation, *k*_B_ stands for the Boltzmann constant, m* is the effective mass, *h* is the Planck constant, and *T* is the temperature. (see Fig. S4a in SI). We also observed such a good correlation for the other composite samples containing a dominant content of CuAgSe β-phase, *i.e.* for Cu_1.2_Ag_0.8_Se and Cu_0.6_Ag_1.4_Se samples (see Fig. S4b and c in the SI). For the Ag_2_Se-rich composite, Cu_0.2_Ag_1.8_Se, a still good qualitative correlation is obtained for m*∼0.22*m*_0_ (see Fig. S4d in the SI). This value corresponds well to the values recently reported for CuAgSe/Ag_2_Se multilayer films.^55^ In the case of a composite with the highest Cu_2_Se content, *i.e.* Cu_1_._6_Ag_0_._4_Se, this approach is clearly inapplicable due to the competitive presence of both p- and n-type carriers with different properties. While the Cu_2_Se component contains a rather high concentration of very heavy holes, dictated by the generation of negatively charged copper vacancies 
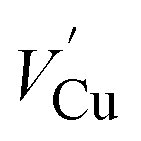
,^56^ the CuAgSe component contributes to the composite with the above mentioned highly mobile electrons of a very low mass. Within the context of the two-carrier model, we will attempt to provide a qualitative explanation of the discrepancy observed between the *S* and *R*_H_ values for this material, considering the dominant carrier mentioned above in each of the participating components using the following equations:5
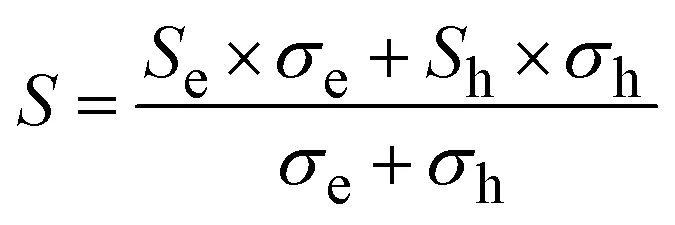
and6
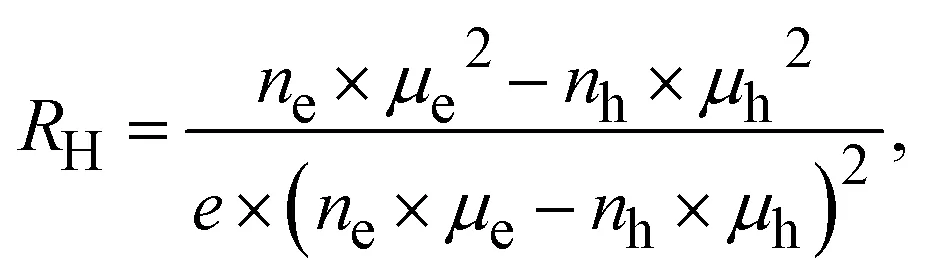
where *S*_e_, *S*_h_, *s*_e_, *s*_h_, *n*_e_, *n*_h_, *µ*_e_, and *µ*_h_ are the Seebeck coefficient (*S*), electrical conductivity (*σ*), carrier concentration (*n*) and carrier mobility (*µ*) for electrons (e) and holes (h), respectively. The observation of very small positive values of *S* in the area of the co-existence of β-phases of Cu_2_Se and CuAgSe, and negative values of *R*_H_ in the same area, can be explained partly also due to the significantly higher effective mass of holes in Cu_2_Se compared to the effective mass of electrons in CuAgSe. This is because the Seebeck coefficient is directly proportional to this value (see eqn (4)). This conclusion can be supported by comparing the *S* values of this sample with those published by the authors^6^ for a material with a slightly lower nominal Cu_2_Se content in a similar composite. The *S* values of the latter material in this region were slightly negative (see the comparison in the inset of Fig. 7b). On the contrary, the negative *R*_H_ values of the material in the area can be explained by the extremely high mobility of light electrons in the CuAgSe component, which predominates over the mobility of heavy holes in the Cu_2_Se component, *i.e.* when *µ*_e_ ≫ *µ*_h_ (see eqn (6)). The predominance is also evidenced from the rather high values of the Hall mobility *µ*_H_ = *R*_H_. *σ* (Fig. 8b), especially for inner members of the studied sample series (*x* = 0.8; 1; 1.4).

For the β-CuAgSe composites with β-Ag_2_Se (*x* = 1.4; 1.8), the *R*_H_ values remain negative, as light electrons predominate in both phases, even after transitioning to the superionic phases. According to the high-temperature X-ray diffractograms of the CuAgSe–Ag_2_Se composite, it consists of the superionic α-phases of both CuAgSe and Ag_2_Se.^6^ This indicates that two types of electrons with similar masses, generated by the formation of mobile cation interstitials (Ag_i_, Cu_i_), dominate electrical transport, resulting in negative *S* and *R*_H_ values in this region. As we previously explained, positive *S* values and negative *R*_H_ values are observed in the superionic α-phase of pure CuAgSe^40^ as a result of the competition between the highly mobile electrons and heavy holes, which are more populous and are generated due to the cation vacancies (V_Ag_, V_Cu_) in the superionic phase.^3^ As for the Cu_2_Se-rich composites, *i.e.* Cu_1.2_Ag_0.8_Se and Cu_1.6_Ag_0.4_Se, the authors^6^ showed that such composites are after the phase transformation of CuAgSe (*T* > 473 K) transformed to a single α-phase material. The aforementioned competitive interplay between the light electrons and the heavy holes persists also in these composites. The concentration of positive holes is observed to increase in a continuous fashion as the content of Cu ions in the cubic α-phase increases. It is evident that while the *R*_H_ values remain negative and approach zero (Cu_1.2_Ag_0.8_Se), or slightly exceed it (Cu_1.6_Ag_0.4_Se), the *S* values are manifestly positive, as is customary for Cu_2_Se-based materials.

**Fig. 9:**
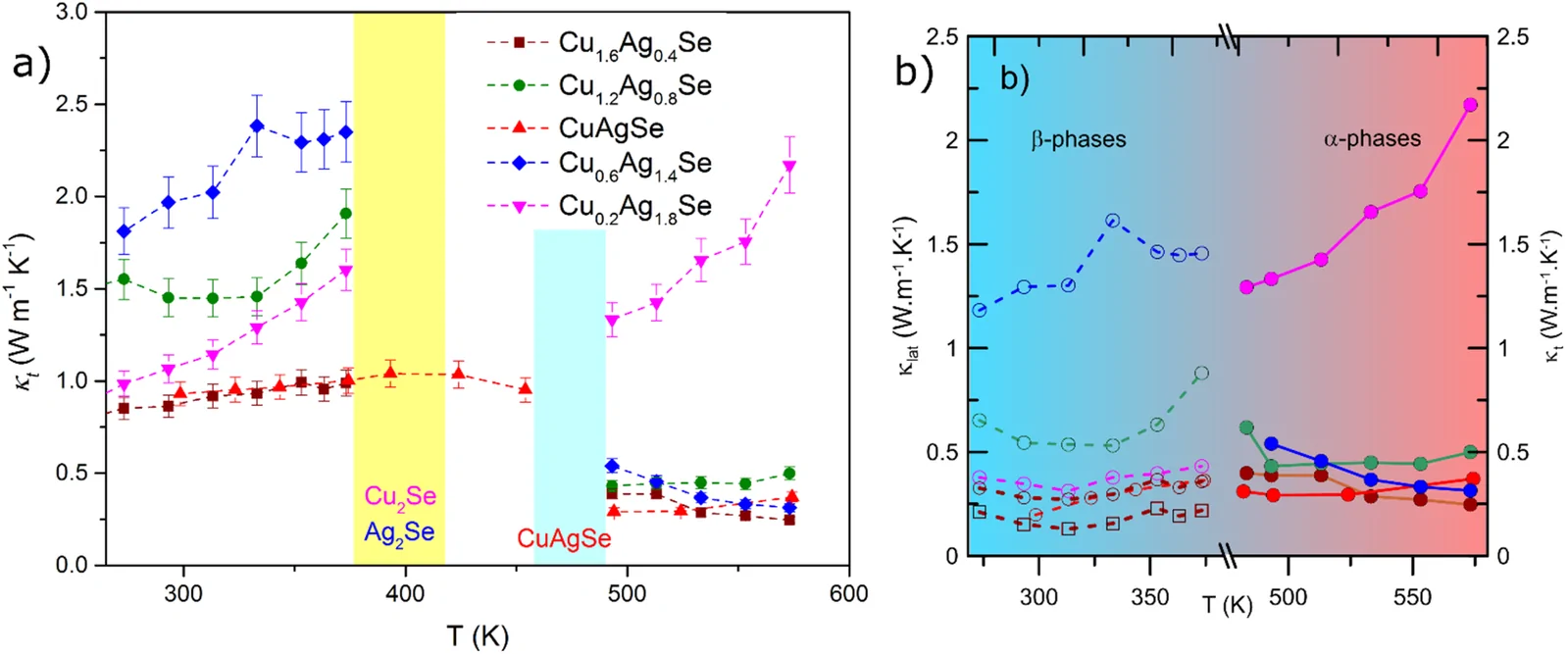
Thermal transport properties of Cu_2−*x*_Ag_*x*_Se samples, temperature-dependent (a) total thermal conductivity, and (b) comparison of the calculated *κ*_lat_-values in the area of the coexistence of β-phases with *κ*_t_-values in the superionic α-phase(s).

Fig. 9a shows the temperature-dependent total thermal conductivity (*κ*_t_). *κ*_t_ values between 1–2 W m^−1^ K^−1^ for all samples increase slightly with temperature up to the β–α transition area of the binary components (Cu_2_Se, Ag_2_Se). After a similar transition for CuAgSe (*T* > 473 K), the values experience a sharp decline, falling below 0.5 W m^−1^ K^−1^.

It is acknowledged that analyzing the thermal conductivity of the composite samples entails a considerable degree of complexity and intricacy. Therefore, a preliminary evaluation of their measured temperature dependencies was conducted. The Wiedemann–Franz law was employed to separate the electronic thermal conductivity (*κ*_el_) and the lattice thermal conductivity (*κ*_lat_) from the total thermal conductivity (*κ*_t_). The following equation was used to calculate *κ*_el_: *κ*_el_ = *L* × *T* × *σ*, where *L*, *T*, and *σ* are the Lorentz number, temperature, and electrical conductivity, respectively. It must be noted that mainly for the composite samples CuAgSe–Cu_2_Se, containing a third contribution to the *κ*_t_ should be considered – the bipolar component of the thermal conductivity *κ*_b_. As follows, *e.g.*, from,^57^ the term can be omitted in the studied high-temperature region (*T* < 570 K), as it becomes non-negligible at rather higher temperatures.

Given the aforementioned good correlation between the experimental values of *S* and *R*_H_ when using the Mott formula for the single-parabolic-band model with acoustic phonon scattering (SPB-APS), we applied the widely accepted^6,58–61^ Kim approximation^62^ to obtain L-values for the majority of our samples in the region of the coexistence of β-phases of the components contained in our composite samples. Thus, the Lorenz number can be approximated, under the assumption that all compositions behave according to the single parabolic band model and are dominated by acoustic phonon scattering, by the following equation: *L* = 1.5 + exp(−*S*/116), where *S* is the measured Seebeck coefficient (in µV K^−1^). Units of L will then be 10^−8^ Ω W K^−1^. The calculated *L*-values for *pure* CuAgSe and composite samples containing Ag_2_Se as the second component ranged from 1.9 to 2 in this temperature range. For the sample containing a small amount of Cu_2_Se in the composite, the value reached 2.1–2.15 in the same temperature range (see Fig. S5 in the SI). As explained earlier, we were unable to ascertain the values for the composite sample with the highest amount of Cu_2_Se. Instead, we used realistic limits for this value (*L* = 2.00–2.44 × 10^−8^ Ω W K^−1^) to calculate *κ*_el_ (see Fig. S6 in the SI). Thus, we were able to separate the thermal conductivity contributions in the studied composites, focusing specifically on the lattice component *κ*_lat_ and how the constituent ratios influence its value. A similar separation of the thermal conductivity contributions in the region following the transition into the superionic α-phase(s) proved to be unreliable and inaccurate. This is primarily due to the presence of a wide variety of point defects and at least two types of free carriers, which complicate the identification of distinct carrier contributions. Therefore, we can only compare the *κ*_el_ values calculated for temperatures below the respective superionic transitions (*T* < 380 K) and the *κ*_t_ values for temperatures above them (*T* > 480 K). As can be seen from this comparison (see Fig. 9b), the values for the CuAgSe and the samples containing Cu_2_Se as the second component in our composites are very similar in both regions, which suggests that *κ*_el_ is very small, and the total thermal conductivity is essentially identical to the lattice contribution in the superionic phase. It should be noted, as mentioned above, that both *pure* CuAgSe and CuAgSe–Cu_2_Se compositions are transformed into single, superionic phase materials^6^ with *κ*_t_ values between 0.25–0.5 W m^−1^ K^−1^. The low thermal conductivity values in the superionic region of these samples are due to two main factors: (a) the high mobility of Cu^+^ and Ag^+^ cations and (b) the use of the ball milling method, which produces a large number of grain boundaries and enhances phonon scattering. Conversely, the samples containing Ag_2_Se-component within the CuAgSe–Ag_2_Se composites in this area displayed characteristics typical of their dominant component. The reason of such behavior is the aforementioned existence of two α-phases, *i.e.* α-Cu_2_Se and α-CuAgSe in the superionic phase. In the sample with an excess of the CuAgSe phase (Cu_0.6_Ag_1.4_Se), the shift to the superionic phase leads to a notable reduction in *κ*_t_, bringing it down to a range of 0.25 to 0.5 W m^−1^ K^−1^. In the sample with an excess of Ag_2_Se (Cu_0.2_Ag_1.8_Se), there is a substantial rise in *κ*_el_, attributed to a significant increase in the concentration of mobile interstitial Ag atoms within the superionic phase, which in turn elevates *κ*_el_.

**Fig. 10:**
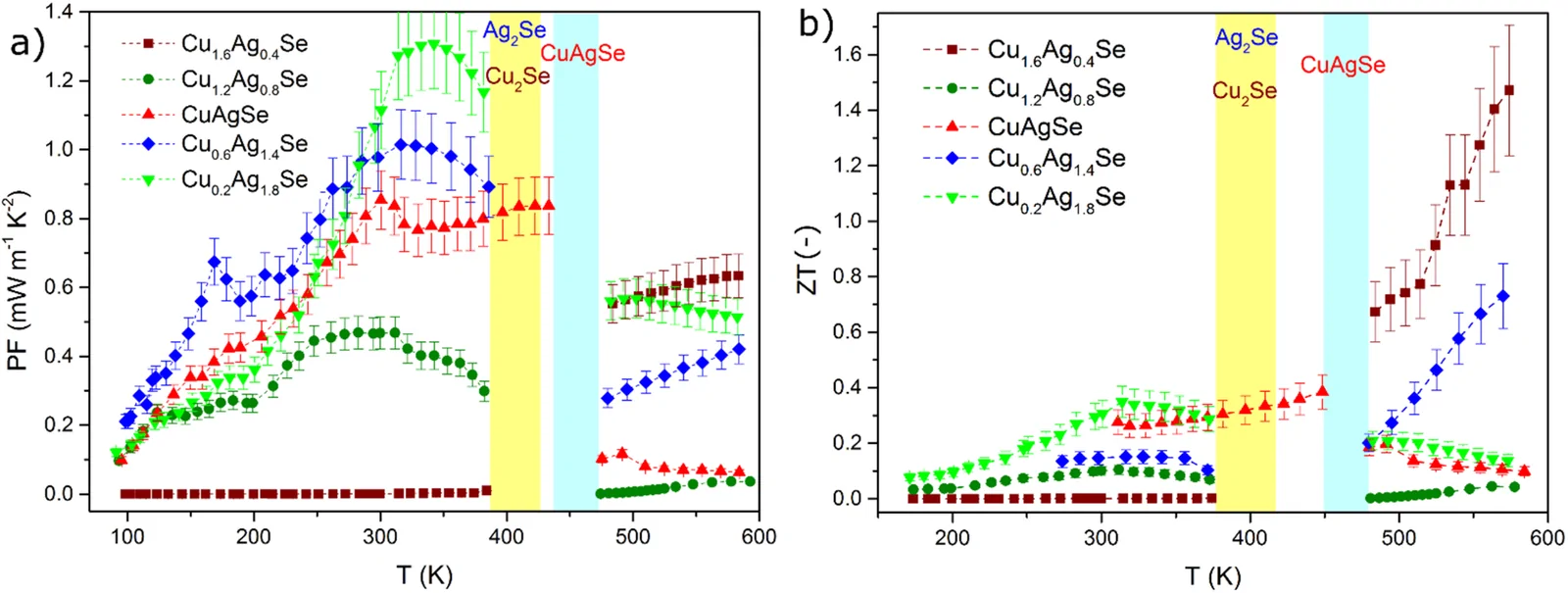
(a) Power factor (*S*^2^*σ*) values, and (b) thermoelectric figure of merit *ZT* as a function of temperature. The phase transition areas of Ag_2_Se, Cu_2_Se, and CuAgSe are highlighted.

The power factor was calculated according to the formula PF = *σS*^*2*^. Its values summarize the influence of electrical transport properties on the material's thermoelectric performance. Thus, its dependencies in Fig. 10a follow the previous trend in electrical conductivity, achieving higher values in the β-CuAgSe region and a decrease in the α-CuAgSe region after the phase transition.

Temperature-dependent *ZT* curves can be seen in Fig. 10b. Some samples reached the highest *ZT* before the phase transition, while others reached it after. *ZT*_max_ are 1.47 @ 574 K for Cu_1.6_Ag_0.4_Se, 0.10 @ 312 K for Cu_1.2_Ag_0.8_Se, 0.38 @ 448 K for CuAgSe, 0.73 @ 570 K for Cu_0.6_Ag_1.4_Se and 0.35 @ 314 K for Cu_0.2_Ag_1.8_Se. The data obtained from thermoelectric measurements are consistent with previously reported trends through the Cu_2−*x*_Ag_*x*_Se compound series. Cu_2_Se doped with Ag was similarly reported to have a nearly zero *ZT* before the phase transition, with a rapid increase above 1.0 after it.^5^ Ag_2_Se is often used as a room-temperature thermoelectric material, reaching maximum *ZT* in the low-temperature region.^36^

**Fig. 11:**
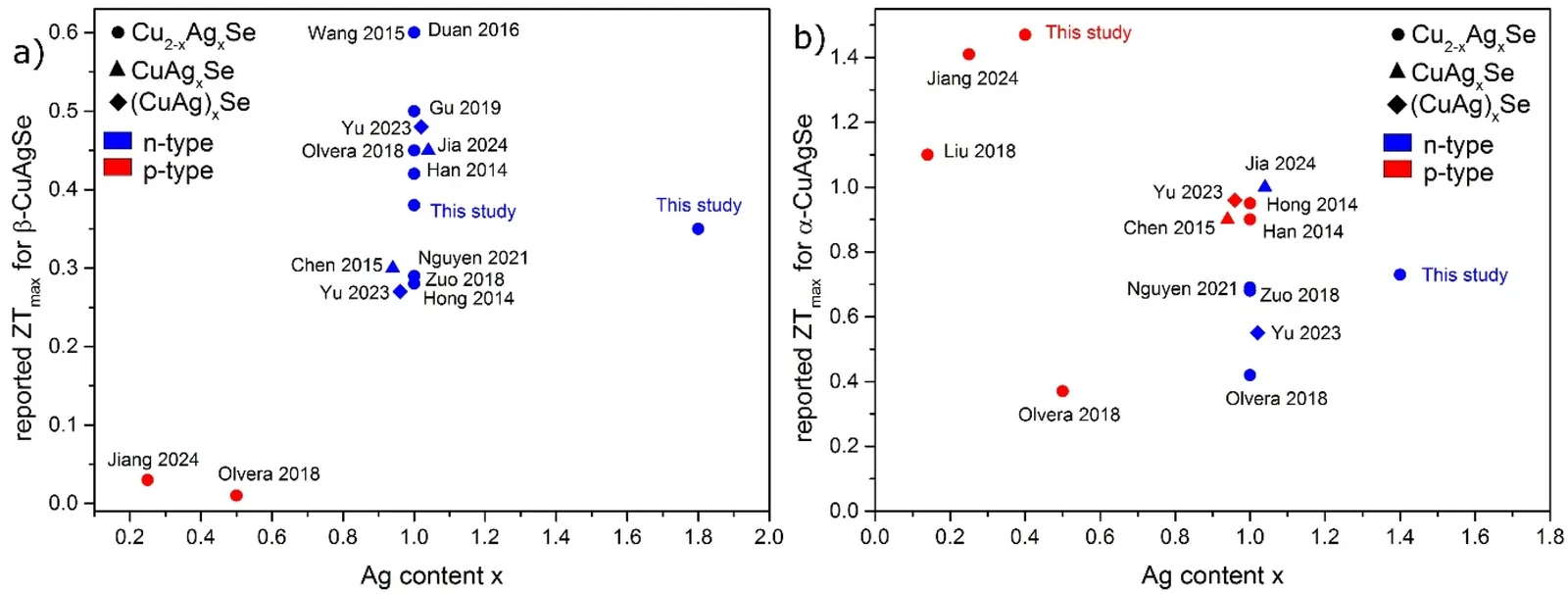
Comparison of reached *ZT*_max_ with reported values in the literature for (a) low-temperature β-CuAgSe and (b) high-temperature α-CuAgSe.^3–6,22,23,25,26,30,33,36,37,63^

**Fig. 12:**
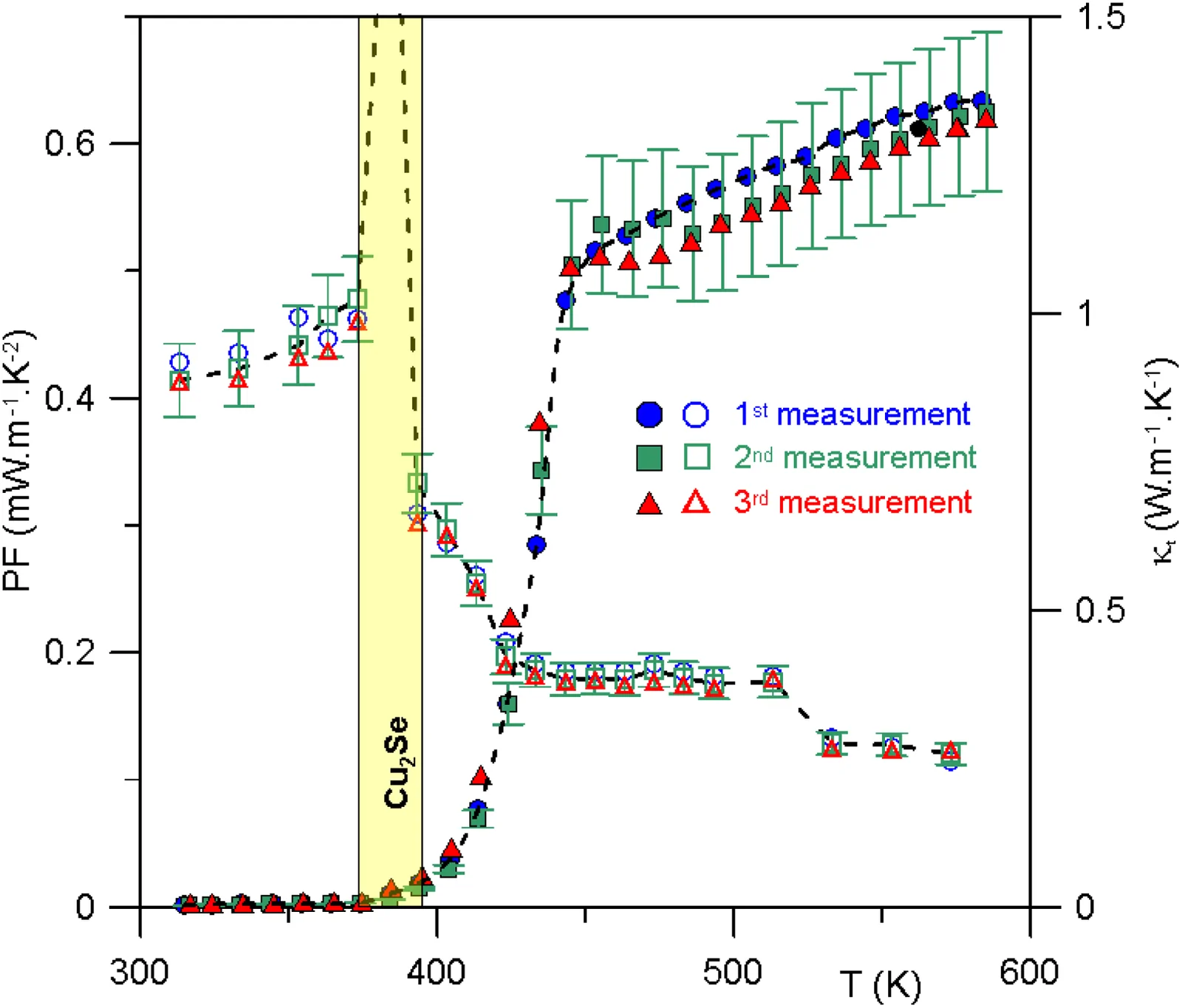
Repeated measurement of electrical and thermal transport properties for the Cu_1.6_Ag_0.4_Se sample, showing reproducibility of the obtained data.

The temperature range in which the material achieves the highest possible *ZT* defines its applicability. Another important factor, from the thermocouple point of view, is whether it is a p-type or n-type semiconductor. In Fig. 11, the best results of this study are presented alongside other published data in two temperature regions: before and after the phase transition. Generally, in the low-temperature region, n-type semiconductors prevail, but in the high-temperature region, p-type semiconductors exhibit better *ZT* values. For β-CuAgSe, below 450 K, the most suitable CuAgSe and Cu_0.2_Ag_1.8_Se were found, with similar *ZT*_max_ values of 0.38 and 0.35 (see Fig. 11a). However, for α-CuAgSe, it appeared more suitable p-type Cu_1.6_Ag_0.4_Se with *ZT*_max_ 1.47 and n-type Cu_0.6_Ag_1.4_Se with *ZT*_max_ 0.73, in this temperature range; our achieved values belonged to the highest (see Fig. 11b). The fairly high *ZT* values of these composites are the result of the contribution of both components. In the aforementioned samples (Cu_1.6_Ag_0.4_Se and Cu_0.6_Ag_1.4_Se), the presence of CuAgSe results in a greater decrease in *κ*_lat_ than with pure Cu_2_Se and Ag_2_Se. Conversely, the presence of excess Cu_2_Se and Ag_2_Se increases the conductivity of the composite compared to pure CuAgSe. The thermal stability of superionic conductors under conditions of high temperature gradient is a matter of particular concern in the context of thermoelectric applications. Fig. 12 illustrates a repeatability test conducted on this sample. The PF and *κ*_t_ values are displayed across three successive cycles. One can see that all data lies within the declared ±10% range for *PF* and ±7% for *κ*_t_.

## Conclusion

Five samples from the Cu–Ag–Se system with varying chemical compositions were successfully synthesized in just 7 min *via* high-energy ball milling. XRD analysis revealed Cu_2−*x*_Ag_*x*_Se as a mixture of ternary CuAgSe and binary selenide of excessing metal, Cu or Ag. In addition, XRD confirmed the presence of the same phases in the samples before and after SPS treatment, with no significant change. XRF analysis clearly showed changes in chemical composition, namely in the Cu : Ag ratio. As a result of the same synthetic conditions for all Cu_2−*x*_Ag_*x*_Se samples, BET surface areas were similarly low, below 0.5 m^2^ g^−1^, and the PSD curves were monomodal, with mean particle sizes *d*(0.5) around 40 µm. In SEM microphotographs of Cu_2−*x*_Ag_*x*_Se sample pellets, using a BSE detector, two phases can be distinguished: a bright phase and a dark phase. Results of chemical analysis of their composition, using the EDX detector, were consistent with the XRD phase analysis. The thermal analysis showed complex behaviour, consisting of two distinct reversible phase transitions, one for every coexisting phase, for most of the Cu_2−*x*_Ag_*x*_Se samples, except for the sample with *x* = 1.0, which had only one phase transition. LSR measurements indicated different types of conductivity in the Cu_2−*x*_Ag_*x*_Se sample series: as the Ag content increased, a progression from p-type over the entire temperature range, through n–p transition, to n-type was observed. According to Hall-effect measurements, two types of charge carriers were identified: before the phase transitions, electrons were dominant; after the phase transitions, holes also participated in electrical transport. CuAgSe together with Cu_0.2_Ag_1.8_Se reached their *ZT*_max_ 0.38 and 0.35 before phase transition, but for p-type Cu_1.6_Ag_0.4_Se, *ZT*_max_ was 1.47@574 K, and for n-type Cu_0.6_Ag_1.4_Se, 0.73@570 K, after phase transition. Thermoelectric properties of Cu_2−*x*_Ag_*x*_Se sample series prepared by rapid and “green” mechanochemical synthesis are comparable to other published results using other synthesis methods.

## Author contributions

Conceptualization, M. A., D. D. and J. N.; formal analysis, D. D., M. B., E. T., T. P., M. B-H. S. Š. and P. R.; investigation, D. D., M. B., E. T., M. B-H., V. P., P. L. and L. B.; writing – original draft, D. D., M. A. and J. N.; writing – review and editing, M. A. and M. B.

## Conflicts of interest

The authors declare no competing interests.

## Supplementary Material

NA-008-D6NA00307A-s001

## Data Availability

Data for this article are available at Zenodo at https://zenodo.org/records/20839932
