# Supplementary material for: Characterization and thermoelectric performance of mechanochemically synthesized Cu–Ag–Se sample series
Source: Nanoscale Adv. 2026 Aug 7;8(17):4957–70. doi: 10.1039/d6na00307a (PMC13471529; doi:10.1039/d6na00307a)
Supplement: Supplementary file 1 [file NA-008-D6NA00307A-s001.pdf]

Supplementary Information (SI) for Nanoscale Advances.
This journal is © The Royal Society of Chemistry 2026

Electronic supplementary material for the paper

Characterization and thermoelectric performance of mechanochemically synthesized Cu - Ag - Se sample series

Dáša Drenčaková, [ab] Marcela Achimovičová, *[a] Jiří Navrátil, [c] Matej Baláž, [a] Erika Tóthová, [a] Tomáš Plecháček, [c] Mária Bali Hudáková, [a] Stanislav Šlang, [c] Viktor Puchý, [d] Petr Levinský, [e] Ludvík Beneš [c] and Pavlína Ruleová [c]

*corresponding author, e-mail: achimovic@saske.sk; tel.: +421557922607

[a] Institute of Geotechnics, Slovak Academy of Sciences, Watsonova 45, Košice, Slovakia

[b] Faculty of Materials, Metallurgy and Recycling, Technical University of Košice, Letná 1/9, Košice, Slovakia

[c] Faculty of Chemical Technology, University of Pardubice, Studentská 573, Pardubice, Czech Republic

[d] Institute of Materials Research, Slovak Academy of Sciences, Watsonova 47, Košice, Slovakia

[e] Institute of Physics, Czech Academy of Sciences, Cukrovarnická 10, Praha, Czech Republic

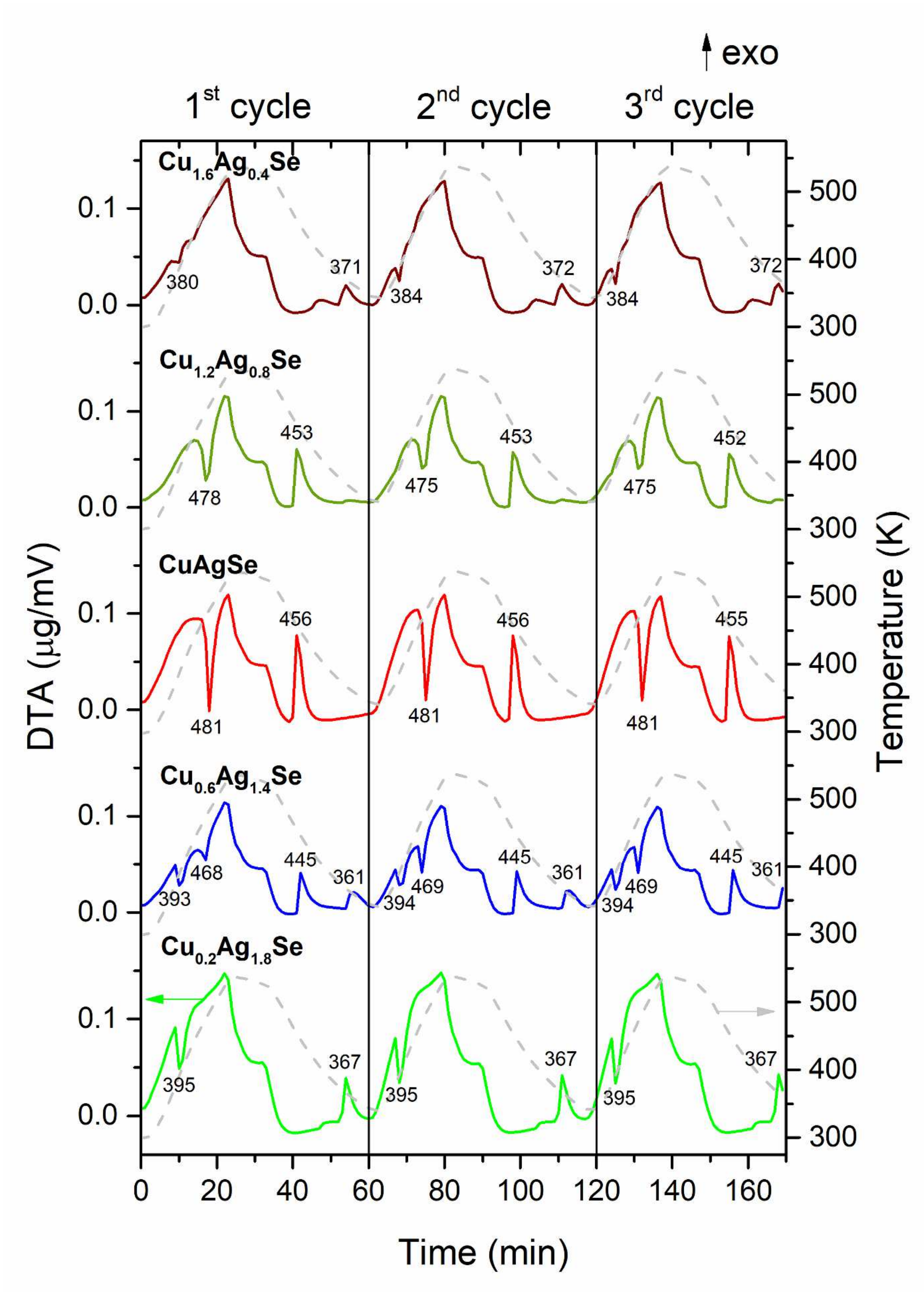

Figure S1. DTA curves of thermal cycling of $Cu_{2-x}Ag_xSe$ samples with highlighted phase transition temperatures during heating and cooling.

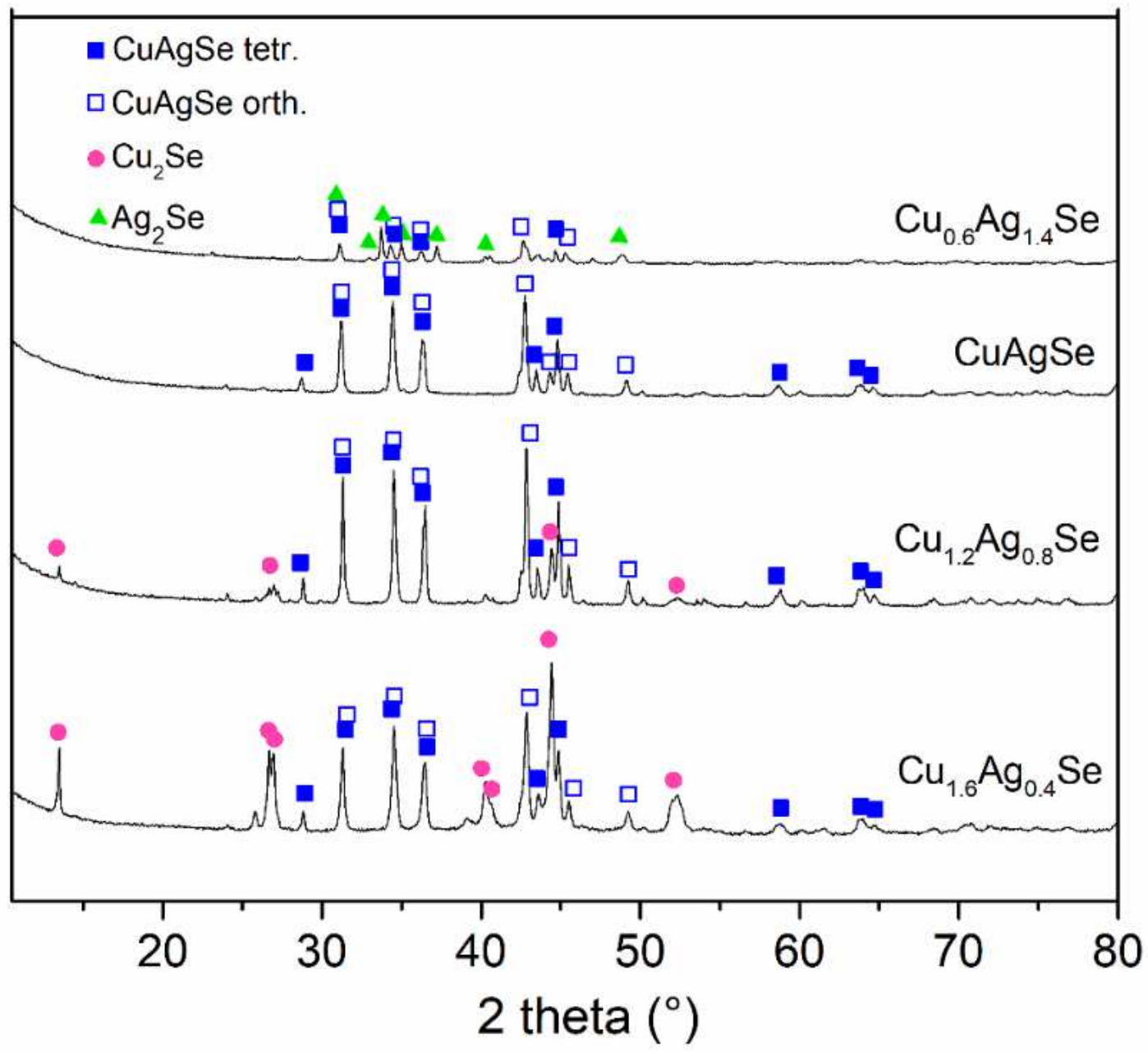

Figure S2. XRD patterns of $Cu_{2-x}Ag_xSe$ after DTA three-fold cycling.

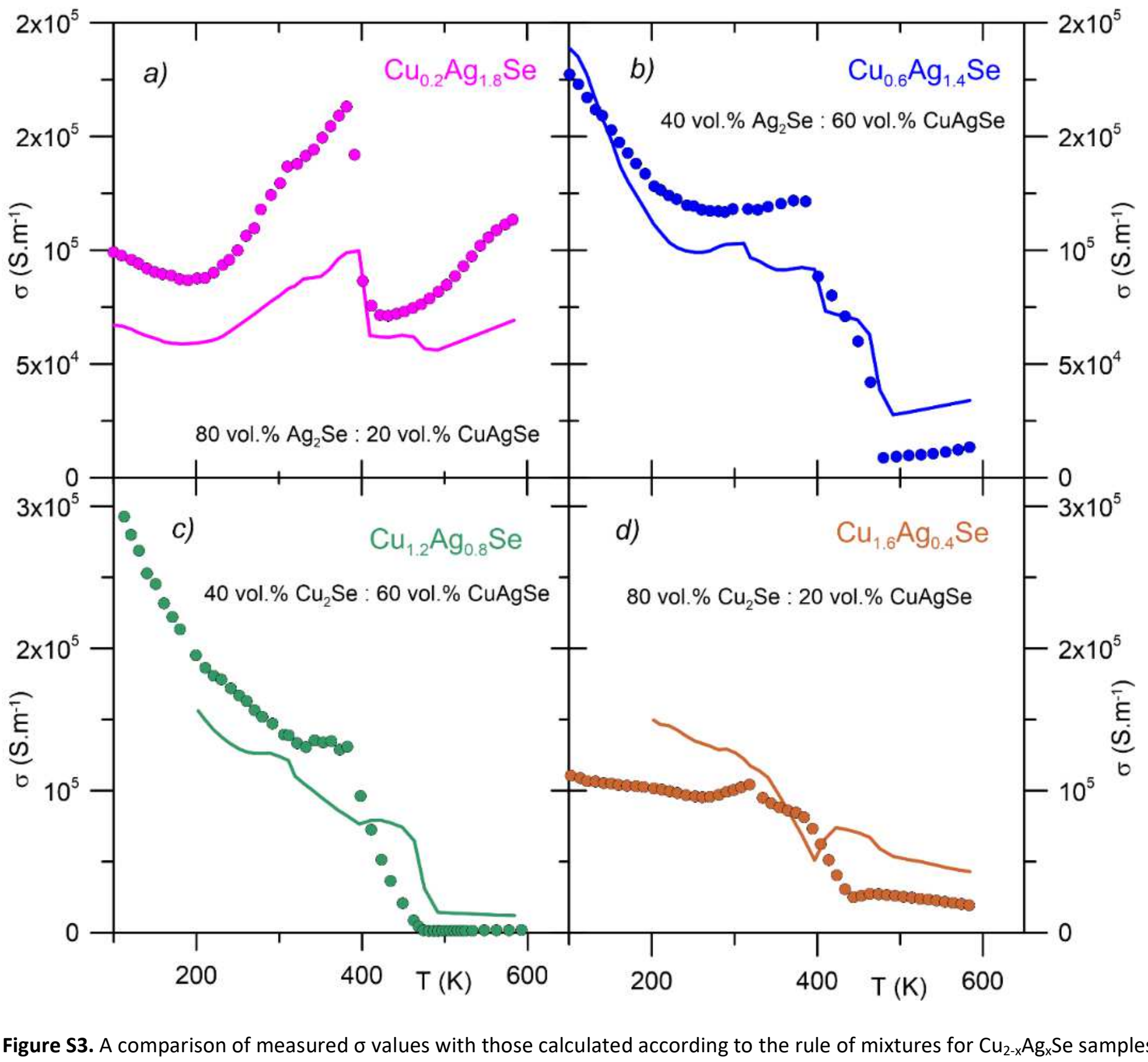

Figure S3. A comparison of measured σ values with those calculated according to the rule of mixtures for $Cu_{2-x}Ag_xSe$ samples.

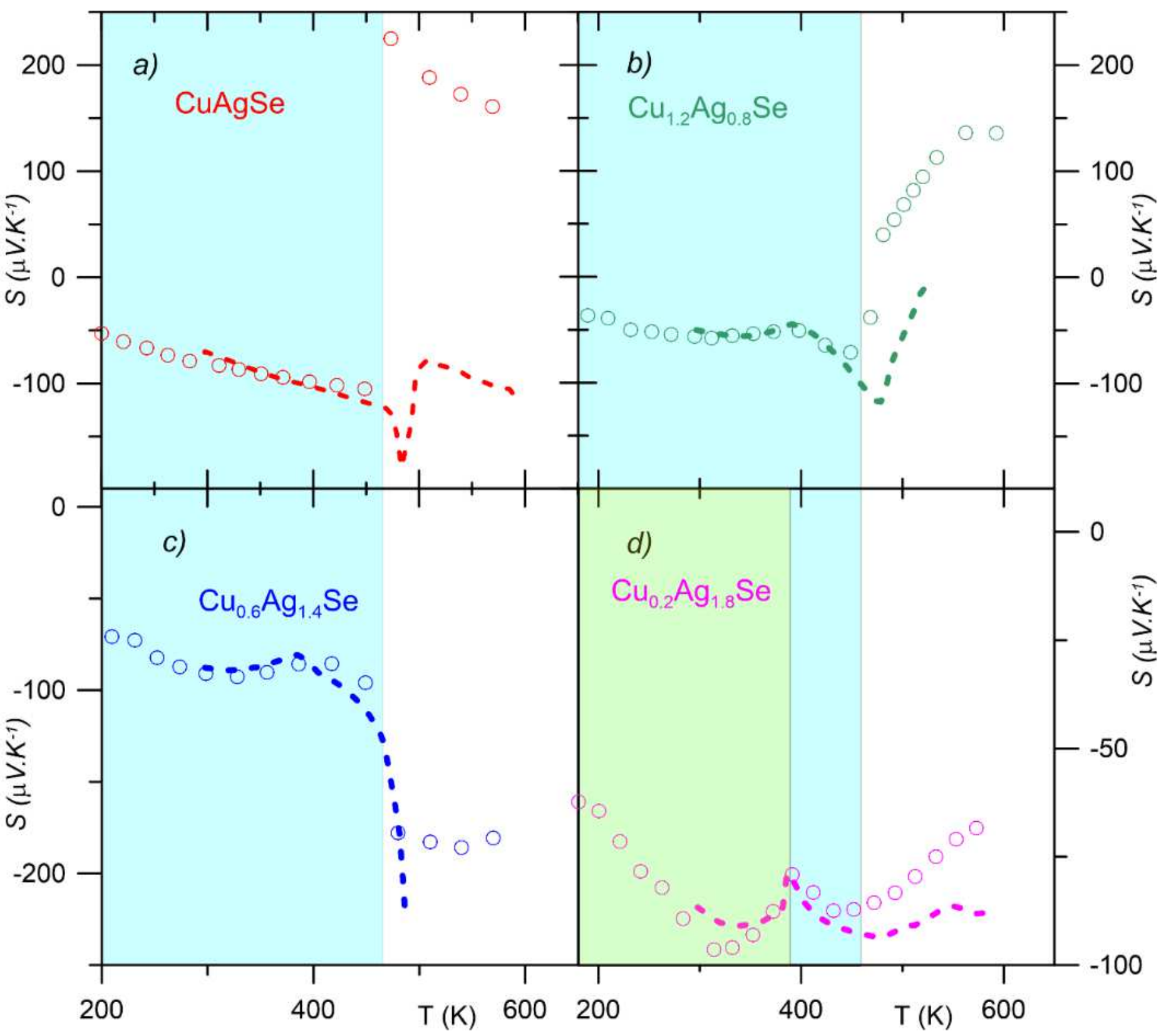

Figure S4. A comparison of the measured Seebeck values of $Cu_{2-x}Ag_xSe$ samples (circles) with those calculated using the Mott formula for the single-parabolic-band model with acoustic phonon scattering (dashed lines). The following effective masses were used to achieve the best fit with the measured values: a) m*=0.095.m_0, b) m*=0.095.m_0, c) m*=0.115.m_0 and d) m*=0.22.m_0,

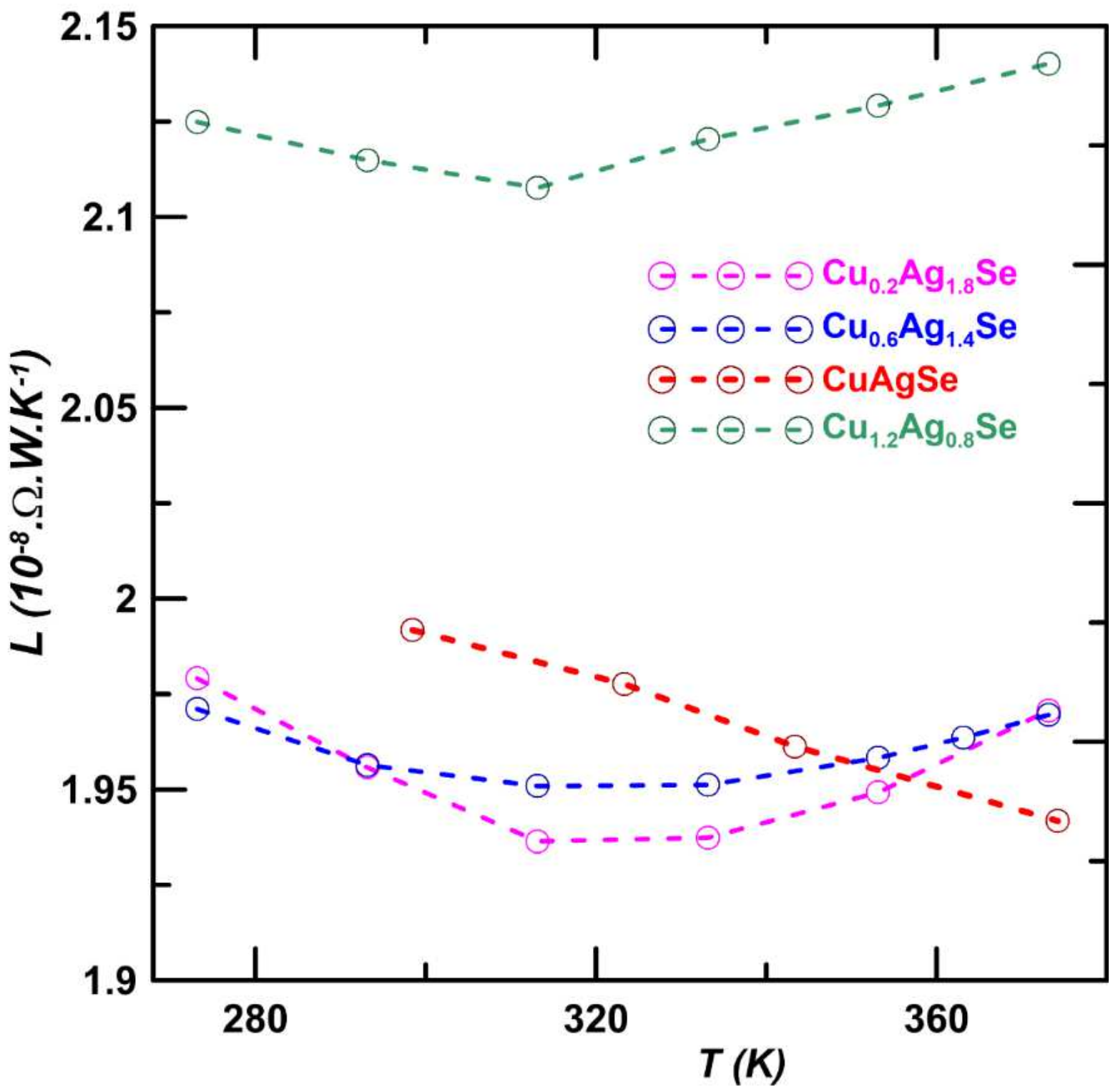

Figure S5. The Lorentz numbers of the $Cu_{2-x}Ag_xSe$ samples calculated using the Kim et al. approximation [62] in the region of coexistence of the β-phases (270-380 K) of the participating components.

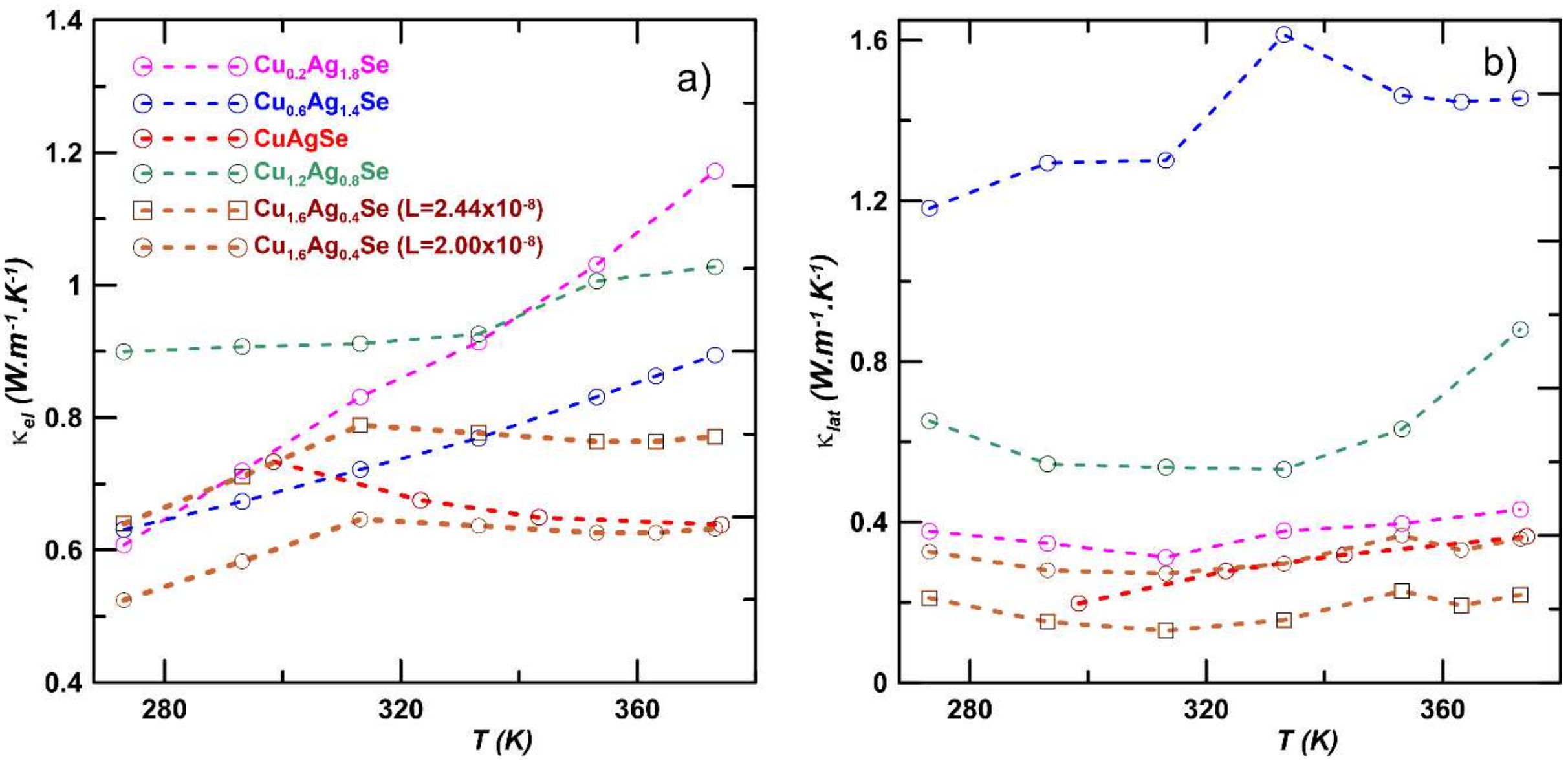

Figure S6. Temperature-dependent thermal transport properties of the calculated values of (a) electronic thermal conductivity and (b) lattice thermal conductivity of $Cu_{2-x}Ag_xSe$ (x = 0.4, 0.8, 1, 1.4, 1.8).
